# Network analysis of surface deformation reveals trunk modularity and synchronization during gait

**DOI:** 10.1371/journal.pcbi.1014388

**Published:** 2026-07-24

**Authors:** Zilu Wang, Jingbang Yang, Yong Wang, Tiantong Wang, Heran Zhong, Fengchen Liu, Chenxi Zhang, Jiangtian Li, Rongli Wang, Ximing Xu, Jiangang Shi, Sunil K. Agrawal, Qining Wang

**Affiliations:** 1 School of Advanced Manufacturing and Robotics, Peking University, Beijing, China; 2 School of Mechatronical Engineering, Beijing Institute of Technology, Beijing, China; 3 School of Rehabilitation Sciences and Engineering, University of Health and Rehabilitation Sciences, Qingdao, China; 4 School of Biological Science and Medical Engineering, Beihang University, Beijing, China; 5 Department of Rehabilitation Medicine, First Hospital, Peking University, Beijing, China; 6 Department of Orthopedic Surgery, Changzheng Hospital, Shanghai, China; 7 Department of Mechanical Engineering, Columbia University, New York, New York, United States of America; 8 Department of Rehabilitation and Regenerative Medicine, Columbia University, New York, New York, United States of America; Dartmouth College, UNITED STATES OF AMERICA

## Abstract

Understanding the dynamic behavior of the human trunk during locomotion is critical for addressing prevalent disorders like low back pain and scoliosis, yet current biomechanical models often oversimplify the trunk as rigid segments. To bridge this gap, we introduce a novel approach combining body surface topography changes with network analysis to characterize trunk motion as a dynamic continuum. By employing the skin surface as a non-invasive observer, we utilized community detection algorithms to identify synchronous deformation regions (SDRs) during gait. Our results suggest that the human back operates as a modular system of synchronized kinematic regions rather than a single homogeneous tissue. These SDRs exhibit robust spatial boundaries and long-range synergies across varying walking speeds, reflecting underlying musculoskeletal dynamics, including spinal kinematics and myofascial coordination. We identified stable clustering in thoracic and lumbar regions, alongside speed-dependent pelvic-scapular synchronization, validating the synergy between upper and lower limb girdles. This framework establishes a methodological foundation for precision rehabilitation by observing the motion of the outer skin. In the future, after validation in larger cohorts and clinical populations, this approach may support candidate surface-derived indicators for evaluating pathological deviations, such as asymmetry in scoliosis or rigid movement patterns in low back pain.

## Author summary

Understanding dynamic trunk function is essential for treating spinal disorders, yet current methods often oversimplify the torso as rigid segments. We introduce a novel framework combining surface topography with network analysis to decode the complex, continuous deformations of the human back during gait. We identified stable synchronous deformation regions that reflect underlying musculoskeletal dynamics, suggesting the trunk as a coordinated, modular system. This non-invasive approach bridges the gap between static anatomy and dynamic function, which may support the development of quantitative surface-derived measures for rehabilitation research.

## 1 Introduction

The human trunk plays a pivotal role in maintaining stability and facilitating the motion of the center of mass during fundamental locomotor tasks, such as walking. However, the biomechanical complexity of the trunk during such movements remains poorly understood, posing a significant challenge for the clinical management of trunk-related disorders. Conditions such as low back pain (LBP) [1] and spinal deformities like scoliosis [2], are prevalent issues that severely restrict mobility and daily activities [3]. While the global burden of these conditions highlights the urgency for effective rehabilitation, a fundamental barrier exists: the lack of high-resolution methods to characterize the trunk motion as a continuous, coordinated system during locomotion.

Current methods for assessing trunk functions struggle to capture the complexity of its motion. Structural imaging modalities, such as MRI [4,5], clearly visualize internal anatomy but are typically restricted to static conditions, failing to account for the functional behavior of tissues during dynamic tasks. While dynamic ultrasound [6] has emerged as a valuable tool for monitoring real-time muscle contractions and fascial gliding, its scope is inherently limited by a narrow field of view, which precludes the observation of large-scale, coordinated patterns across the entire dorsal surface. To address the need for functional mechanical characterization, advanced robotic platforms, such as the Robotic Spine Exoskeleton (RoSE), have been developed to quantify the three-dimensional stiffness of the human torso *in vivo*, providing insights into the biomechanical differences between healthy individuals and those with spinal deformities [7,8]. Complementary to these experimental measures, biomechanical approaches often rely on musculoskeletal (MSK) modeling to characterize dynamic abnormalities [9]. However, the complex structure of the trunk—comprising multiple vertebrae and non-osseous tissues—poses a unique challenge. To facilitate calculations, the trunk is typically simplified into rigid body segments (e.g., thorax and pelvis) [10]. This simplification overlooks the continuous surface deformations and intervertebral motions that occur during walking, leading to discrepancies between computational models and *in vivo* behavior [11]. While techniques like rasterstereography have attempted to reconstruct topographical features [12,13], a comprehensive dynamic representation of the trunk’s surface topography during cyclic tasks remains largely unexplored.

In this study, we introduce a novel technical approach combining “body surface topography changes” with “network analysis” to characterize trunk motion. The human dorsal skin is not merely a passive covering but a complex mechanical interface coupled with the underlying myofascial system. The topographic fluctuations, though subtle, aggregate the collective output of the trunk’s deep-seated biological structures. By employing the skin surface as a non-invasive observer of the underlying complex musculoskeletal dynamics, we utilized community detection algorithms to identify and quantify “synchronous deformation regions” (SDRs) during gait. Our findings suggest that, in this proof-of-concept study, SDRs exhibit robust spatial boundaries and long-range synergies that persist across varying walking speeds. These regions are indicative of underlying myofascial and musculoskeletal coordination. This approach aims to establish a methodological foundation for the quantitative rehabilitation assessment and may be applied for precise interventions of the trunk disability in the future.

## 2 Results

### 2.1 Dynamic deformation of dorsal skin during walking

**Fig 1:**
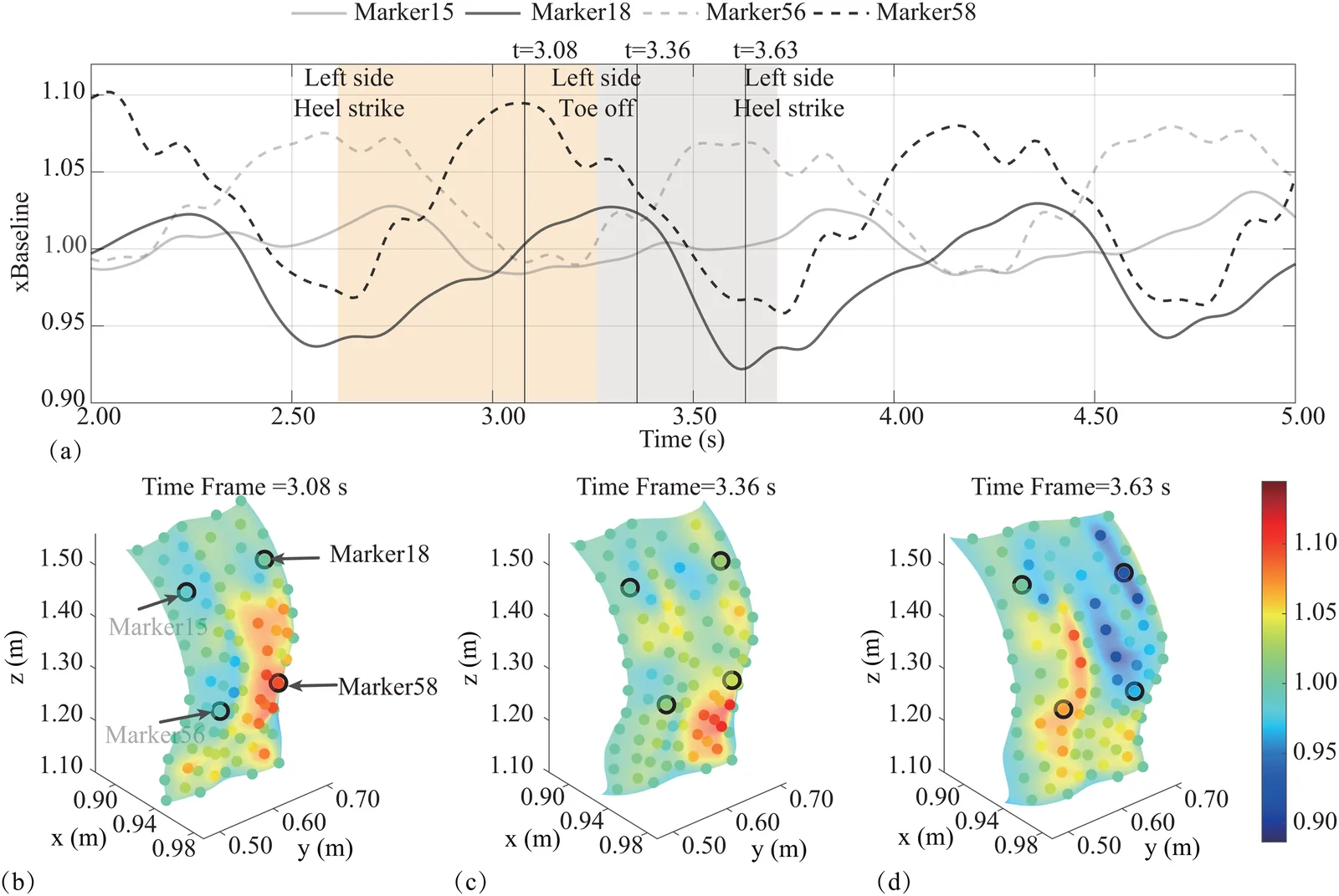
Dynamic deformation of dorsal skin during walking. **(a)** The normalized neighborhood areas for four selected markers (Markers 15, 18, 56, 58) at a walking speed of 1.3 m/s. The curves demonstrate clear periodicity synchronous with the gait cycle, indicated by the moments of leftside heel strike and leftside toe off. The different times at which the same-side markers (e.g., Marker 58 vs. Marker 56) reach the peak of deformation reflect the inconsistency in the phase of skin deformation. **(b–d)** Skin deformation across the dorsal surface at three specific time frames: *t* = 3.08 s (left stance), *t* = 3.36 s (post left toe off), and *t* = 3.63 s (early left heel strike). The color bar indicates normalized neighborhood areas, where red (values >1) represents extension and blue (values <1) represents compression.

Fig 1 presents the temporal and spatial deformation characteristics of the dorsal skin at a walking speed of 1.3 m/s. The analysis of local area changes reveals that dorsal skin deformation is not a uniform and passive phenomenon but rather a complex, rhythmic mechanical response closely coupled with the gait cycle.

As depicted in Fig 1a, the variations in the normalized neighborhood area (**Methods**), which represents the local skin stretch ratio, exhibited a clear periodicity synchronous with the gait cycle. The dynamic behavior of selected markers (Markers 15, 18, 56, and 58) demonstrated cyclic motion, consistently aligning with the alternating gait phases. Overall, the deformations of the left and right skin surfaces, delineated by the spinal midline, are in anti-phase. For example, the rightside skin (Markers 18 and 58) experienced progressive stretching throughout the left foot’s stance phase (leftside heel strike to leftside toe off). Subsequent to leftside toe off, and continuing through the left swing phase, this skin segment gradually contracted. This confirms that the mechanical behavior of the dorsal skin is functionally integrated with the kinematic chain of gait.

Despite the global periodicity, a distinct phase inconsistency was observed among individual markers, indicating the presence of heterogeneous deformation regions. The peak stretch values for different anatomical locations were not attained simultaneously, even for the ipsilateral skin surface. For instance, Marker 58 (situated on the lower right back) reached its maximum extension at approximately *t* = 3.08 s, whereas Marker 18 (situated on the upper right back) reached maximum displacement at around *t* = 3.36 s. This temporal lag suggests that the skin operates through a coordinated sequence of regional deformations rather than a monolithic expansion.

The spatial distribution of skin deformation, visualized in the 3D topographical maps (Fig 1b–d), further elucidates a collaborative mechanism between the left and right dorsal regions. During the rightside swing phase (around *t* = 3.08 s), the skin on the right side exhibited significant extension (indicated by red zones, stretch ratio > 1.05), while the contralateral leftside remained relatively neutral or compressed. During the leftside swing phase (around *t* = 3.63 s), *t*he deformation pattern reversed: the left dorsal region (around Marker 56) underwent maximum stretching, while the right side transitioned into a compression phase (indicated by blue zones, stretch ratio < 0.95).

### 2.2 Identification of synchronous deformation regions

**Fig 2:**
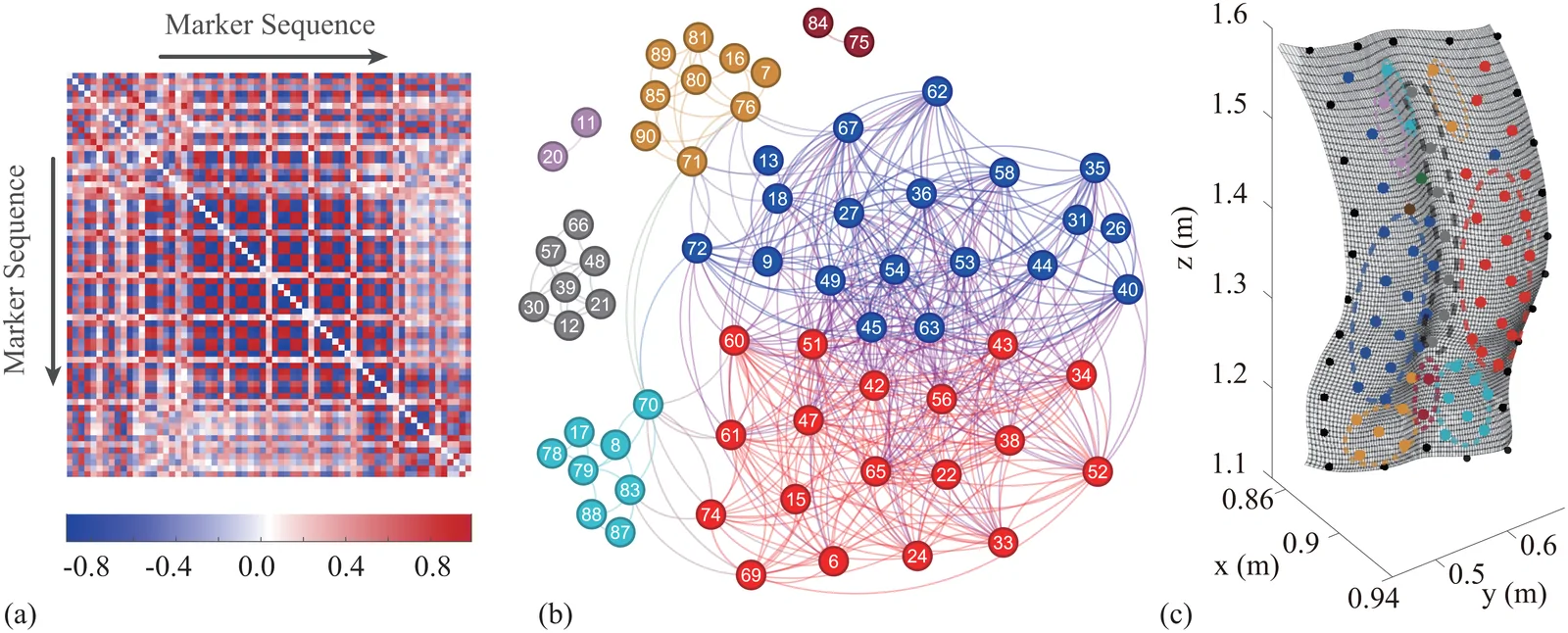
Identification of SDRs via network analysis. **(a)** Spearman correlation matrix heatmap of the normalized neighborhood area variation for all marker pairs. The matrix exhibits a clear block-diagonal structure, signifying the existence of distinct synchronous regions. High positive correlation is shown in red, and strong negative correlation in blue. **(b)** Correlation signed network derived from the correlation matrix. Edges with a correlation coefficient absolute value $\left|\rho_{ij}\right|<0.8$ were pruned to isolate strong synergistic coupling. Community detection was used to segment the network into functional clusters (indicated by color-coding). **(c)** Spatial mapping of the identified functional communities to the dorsal marker array. Connected regions are indicated by dashed lines of the corresponding colors.

To quantify the spatial coordination of dorsal skin deformation, we analyzed the functional connectivity between markers based on their neighborhood area variations. Fig 2a displays the heatmap of the pairwise Spearman correlation coefficients ($\rho_{ij}$) for all marker pairs (*Methods*). The matrix exhibits a block-diagonal structure with distinct regions of high positive correlation (red) and high negative correlation (blue), suggesting that the dorsal skin can be functionally segmented into discrete regions that deform synchronously or are in anti-phase.

To extract SDRs, a correlation signed network was constructed by filtering the correlation matrix with a strict threshold (*h* = 0.8). Edges representing weak correlations ($\left|\rho_{ij}\right|<h$) were pruned, resulting in an undirected graph where connections represent only strong kinematic coupling. Subsequent community detection analysis applied to this network revealed a modular structure with modularity *Q* = 0.250 (details in *Methods*), as shown in Fig 2b.

Within the detected communities, a sub-segmentation based on the sign of the correlation edges was performed. It was observed that the identified communities were balanced in nature, allowing for a clear separation into sub-regions based on synchronous (positive) versus anti-phase (negative) deformation. This network topology highlights specific mechanical couplings. Specifically, inter-community edges linking the red and blue modules, as well as specific local connections involving Node 70 (i.e., edges {70, 71}, {70, 72}, and {70, 76}), are characterized by negative correlation weights. This anti-phase relationship suggests a compensatory deformation mechanism, where the expansion within one functional domain occurs in tandem with the localized contraction of its counterpart.

Fig 2c maps these network communities back to the physical geometry of the dorsal surface. The spatial clustering at this speed reveals a complex, multi-regional coordination pattern: The two largest clusters (red and blue) occupy the mid-to-lower dorsal regions on the right and left sides, respectively. These correspond to the zones of maximum anti-phase deformation driven by the gait cycle. A distinct functional cluster (grey) was uniquely identified along the midline of the spine. This zone forms a coherent kinematic boundary separating the lateral deformations, likely reflecting the relative stability of the vertebral column amidst the dynamic stretching of the paravertebral skin. The existence of spatially disjoint functional communities (orange and cyan) is a unique feature, which indicates the presence of long-range kinematic synergies. The orange cluster aggregates markers from disparate regions, bridging the lower-left and upper-right dorsal zones. Similarly, the cyan cluster connects the lower-right with the upper-left regions. These “diagonal” configurations suggest that the dorsal skin operates as a global mechanical continuum, coordinating deformation across non-adjacent regions (e.g., contralateral shoulder and hip regions) in response to the cross-body torque transfer inherent in high-speed walking.

### 2.3 Consistency of functional segmentation across locomotor demands

**Fig 3:**
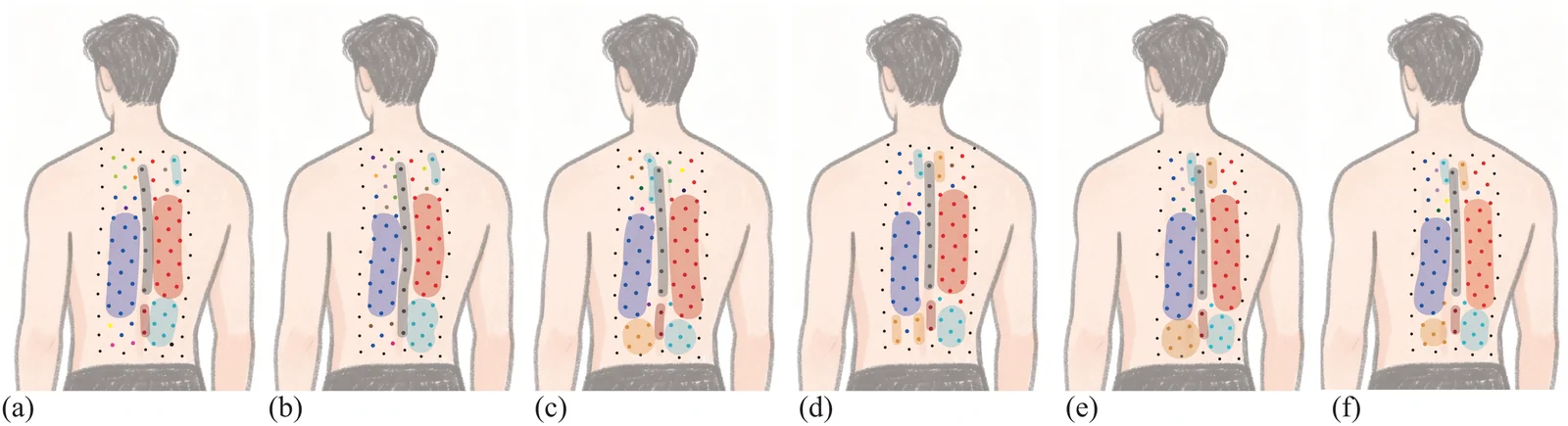
The SDRs at different walking speeds. **(a)** The regions at speed 0.7 m/s. **(b)** The regions at speed 0.9 m/s. **(c)** The regions at speed 1.1 m/s. **(d)** The regions at speed 1.3 m/s. **(e)** The regions at speed 1.5 m/s. **(f)** The regions at speed 1.7 m/s.

To investigate the evolution of dorsal skin deformation patterns and verify the robustness of the identified functional structures, we extended the network analysis across six distinct walking speeds, ranging from 0.7 m/s to 1.7 m/s (in 0.2 m/s increments). Fig 3a–3f illustrates the spatial mapping of the functional communities identified at each speed level. The results demonstrate a remarkable topological stability in the core functional organization. Across the entire speed spectrum, the two dominant, anti-phase communities (red and blue) remained largely invariant in their spatial boundaries and position, confirming the left-right reciprocal deformation as the fundamental kinematic mode of the dorsal skin during gait. The cluster identified along the spinal midline (grey), serving as the kinematic boundary, was consistently present across all tested speeds. The invariance of these functional community structures indicates that the detected patterns reflect stable underlying mechanical coordination strategies that the musculoskeletal system employs to manage skin tension and trunk stability, demonstrating strong robustness against changes in walking speed. Furthermore, the prevalence of long-range synchronous domains (orange and cyan) became gradually apparent with increasing velocity, first emerging at 1.1 m/s and stabilizing into consistent features at high speeds (i.e., above 1.3 m/s), indicating enhanced coordination in regional skin dynamics. Their repeated appearance at higher walking speeds further supports interpreting these multi-marker, spatially disjoint communities as structured long-range synergies rather than as isolated noisy fragments. Robustness analyses for threshold selection, reduced marker layouts, and 5-s moving windows are provided in S1 Text.

## 3 Discussion

In this study, we combined high-density reflective marker tracking with network analysis to quantitatively characterize the spatiotemporal deformation of dorsal skin during gait. By analyzing these interconnected deformation patterns, our results suggest that the human back operates as a modular system of synchronized kinematic regions rather than a homogeneous sheet of tissue. The emergence of stable, spatially coherent clusters across multiple walking speeds suggests that body surface topography effectively encodes the underlying interplay of spinal motion, muscular engagement, and fascial tension. This discussion focuses on decoding these surface patterns to elucidate their anatomical origins, the physiological mechanisms governing their consistency, and their potential as candidate indicators for future studies of trunk dysfunction.

### 3.1 Anatomical and functional interpretation of identified clusters

Although the community detection analysis identified multiple clusters, the present study primarily focuses on those with strong anatomical correlations and functional significance.

First, clusters representing spinal movement (grey) were consistently observed and remained stable across all six walking speeds. This stability can be attributed to the close proximity of the skin to the underlying spinous processes in this region [14]. Since the overlying skin and superficial fascia are mechanically constrained near the midline, this region is less free to undergo the lateral viscoelastic deformation observed in the paraspinal regions. Consequently, changes in the surface area of the skin are highly correlated with vertebral kinematics. Notably, at five of the six tested speeds, these spinal clusters were clearly partitioned into distinct thoracic and lumbar regions. This separation likely arises because, during actual gait, the kinematic synchronization among vertebrae within the same region (i.e., thoracic-thoracic or lumbar-lumbar) exceeds the synchronization between these two distinct regions.

**Fig 4:**
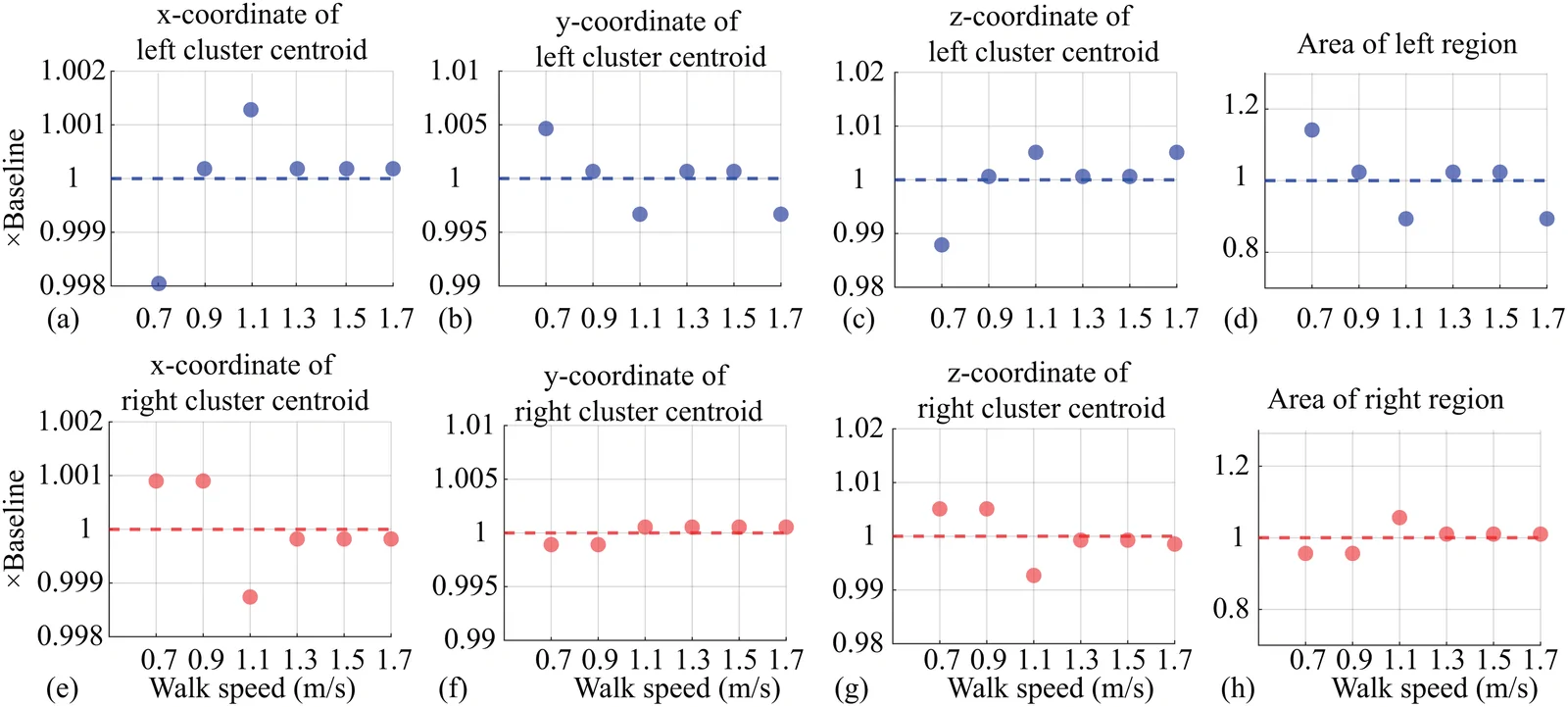
Robustness of the SDRs across gait speeds. **(a–h)** Analysis of the clusters’ geometric properties across various walking speeds (0.7 m/s to 1.7 m/s). The dashed line at 1.00 denotes the within-subject mean value across all six tested walking speeds for the corresponding parameter. **(a, e)** Normalized *x*-coordinate (anterior-posterior position) of the left and right cluster centroids. **(b, f)** Normalized *y*-coordinate (left-right position) of the left and right cluster centroids. **(c, g)** Normalized *z*-coordinate (vertical position) of the left and right cluster centroids. **(d, h)** Normalized area of the left and right clusters. The small variation in centroid location and area demonstrates the high robustness of the functional segmentation methodology to changes in gait speed.

Second, the two most extensive clusters (red and blue) were situated roughly over the (left and right) lower dorsal thoracic regions, extending down to the upper lumbar segments. These clusters exhibited topological stability across all six speeds (Fig 4), maintaining remarkably similar spatial distributions and boundaries. The emergence and stability of these large lateral regions may be associated with the nature of human gait as a complex, three-dimensional coupled movement. Specifically, the flexion-extension cycle induces a generalized stretching of the dorsal integument. In the coronal plane, lateral flexion recruits the ipsilateral erector spinae and quadratus lumborum, where mechanical forces are transmitted to the surface via the thoracolumbar fascia, generating longitudinal tension bands [15,16]. Simultaneously, axial rotation engages the Posterior Oblique Subsystem—functionally linking the latissimus dorsi and the contralateral gluteus maximus—and follows the myofascial continuity described as the ‘Back Functional Line’ [17,18]. Markers situated within this specific region undergo synchronous, in-phase stretching and compression during the gait cycle. In our mathematical framework, this localized phase consistency translates directly into high positive rank correlation coefficients ($\rho_{ij}>0.8$).

Finally, distinct clusters emerged around the posterior superior iliac spines (PSIS) bilaterally. These regions were observed exclusively at higher walking speeds (≥ 1.1 m/s), while indiscernible at lower speeds (< 1.1 m/s). The appearance of these regions may relate to the interaction between the ilium and the overlying skin surface. Higher walking speeds necessitate significantly larger pelvic rotation [19], resulting in substantial topographical changes; thus, the synchronization of skin surface contraction becomes sufficiently pronounced to be detected. This increased clarity of musculoskeletal dynamics at higher velocities is further reflected by another significant phenomenon: the emergence of long-range synchronization between these pelvic clusters and the superior angle of the respective contralateral scapulae at speeds ≥ 1.1 m/s. During gait, the scapula undergoes reciprocating upward and downward rotation, and interactions between its prominent edges and the skin induce topographic surface changes. Intriguingly, these small scapular regions demonstrated a remote synchronization phenomenon with the spatially disjoint larger clusters below. This finding likely reflects the high degree of synergy in dorsal kinematics: just as forward arm swing coordinates with contralateral leg flexion, the scapula and contralateral ilium—representing the pectoral and pelvic girdles, respectively—follow a similar movement paradigm. By projecting three-dimensional skeletal motion onto two-dimensional skin surface deformation patterns, this study enables the comparison of upper and lower limb girdle mechanics within a unified dimension. The emergence of these discrete, synchronized clusters serves as collateral evidence for the synergy between these two systems.

### 3.2 Physiological justification for spatial consistency

In characterizing the SDRs, we used spatial support as an important plausibility criterion, but not as a strict requirement of physical contiguity. Because the dorsal skin behaves as a continuous viscoelastic field [20,21], direct deformation transmission is expected to generate locally coherent regions [22,23]. At the same time, the correlation network quantifies statistical co-variation over time rather than direct material adjacency. Therefore, an SDR may appear either as a contiguous local domain or as a multi-marker, spatially disjoint pattern when remote sites are driven by the same gait-synchronous mechanical mode. On this basis, we distinguished between (i) internally coherent cross-regional communities, such as the orange and cyan clusters in Fig 2c, which were retained as long-range synergies, and (ii) isolated single markers or tiny fragments lacking clear spatial support, which were not interpreted as SDRs and were treated cautiously as likely artifacts or computational noise.

### 3.3 Comparison with traditional sparse configurations

Traditional approaches for assessing trunk kinematics predominantly rely on sparse sensor configurations, such as inertial measurement units (IMUs) or surface electromyography (sEMG). While IMUs are highly effective in quantifying global dynamics—including trunk inclination, joint angles, and spatiotemporal gait parameters—they typically treat the torso as a series of rigid segments, thereby masking the complex, localized deformation patterns of the dorsal surface. Similarly, although sEMG can characterize functional synergies between specific muscle groups through electrical activity [24–26], its spatial resolution is inherently limited by the discrete placement of electrodes, making it localized rather than topographic.

In contrast, our framework transcends the “rigid-body” assumption by treating the dorsal skin as a dynamic continuum. By employing a dense network of 95 markers, we achieve a high-resolution mapping of surface topography that is mathematically invisible to sparse sensing techniques. This approach not only identifies SDRs as distinct functional domains but also captures long-range spatiotemporal correlations across the entire trunk. Such macroscopic insights into the collective coordination of the musculoskeletal system provide an additional layer of information and may support the development of candidate surface-derived indicators for trunk synergy and potential pathological deviations.

### 3.4 Potential clinical implications and therapeutic relevance

If validated in larger clinical cohorts, the quantification of SDRs may provide candidate indicators for precision rehabilitation research. Specifically, for patients with mild scoliosis, calculating the location and extent of these convergent regions may help characterize asymmetry associated with scoliotic trunk mechanics. This spatial mapping facilitates the development of targeted intervention strategies, such as localized paraspinal myofascial release and specific neuromuscular training, to address asymmetric loading patterns [27,28]. Furthermore, in the context of low back pain (LBP), the body often adopts a “protective guarding” strategy, where nociceptive input significantly constrains spinal flexibility and degrees of freedom. Consequently, LBP patients may exhibit a broader area of functional convergence, reflecting a rigid, en bloc movement pattern [29]. As the pathology evolves, the surface area and distribution of these structures are likely to shift, serving as a dynamic indicator of disease progression or recovery. Therefore, longitudinal monitoring of these regional variations could be explored as a candidate metric for tracking functional changes. Ultimately, accumulating such quantitative data across larger cohorts and repeated sessions may help clarify the mechanisms of trunk functional disorders and inform data-driven therapeutic strategies.

### 3.5 Limitations and future work

The most important limitation of this study is its single-participant and single-trial design. Although the analyzed walking segments contained multiple gait cycles and the SDR patterns were examined across six walking speeds, the present dataset does not allow estimation of inter-subject variability, test-retest reliability, or population-level generality. Therefore, the SDRs identified here should be interpreted as within-participant proof-of-concept patterns rather than universal anatomical modules or validated clinical biomarkers.

A second limitation is that the present participant was a healthy adult male. The SDR organization observed in this study may differ in individuals with different body morphology, gait strategy, age, sex, or trunk-related disorders. In particular, the diagnostic relevance of SDRs for scoliosis, low back pain, or other trunk motor dysfunctions remains unvalidated. Future studies should include larger cohorts, repeated trials across sessions, and clinical populations to determine whether the proposed network-derived measures are reproducible, sensitive to pathology, and specific to different functional impairments.

In addition, the surface-marker method observes external dorsal-surface motion and cannot fully separate vertebral rigid-body motion, fascial transmission, muscle activity, and soft-tissue deformation. Future work should combine the present surface-topography framework with complementary measurements, such as dynamic imaging, low-dose biplanar radiography, ultrasound, or electromyography, to validate the anatomical sources of the observed SDRs. These extensions will be necessary before the proposed framework can be used as a clinically validated biomarker for trunk function.

## 4 Conclusion

This study presents a proof-of-concept framework integrating body surface topography with network analysis to characterize dorsal surface deformation during gait. In one healthy participant, the framework identified distinct anti-phase deformation domains with stable spatial boundaries across walking speeds and speed-dependent scapular-pelvic synchronization patterns. These findings suggest that marker-derived dorsal surface deformation can provide a non-invasive observer for coordinated trunk motion.

However, the present results should be interpreted as within-participant feasibility evidence rather than population-level validation. Larger cohorts, repeated trials, and clinical populations are required to determine whether SDRs are reproducible across individuals and whether they can serve as clinically useful indicators of trunk dysfunction. With such validation, the proposed framework may contribute to future quantitative assessment and personalized rehabilitation of musculoskeletal conditions such as scoliosis and low back pain.

## 5 Materials and methods

### 5.1 Ethics statement

This research protocol received formal approval from the Local Ethics Committee of Peking University First Hospital (under Application No. 2022#429–002) following strict adherence to research ethics, and written informed consent was obtained from the subject.

### 5.2 Mechanisms of dorsal surface deformation during locomotion

**Fig 5:**
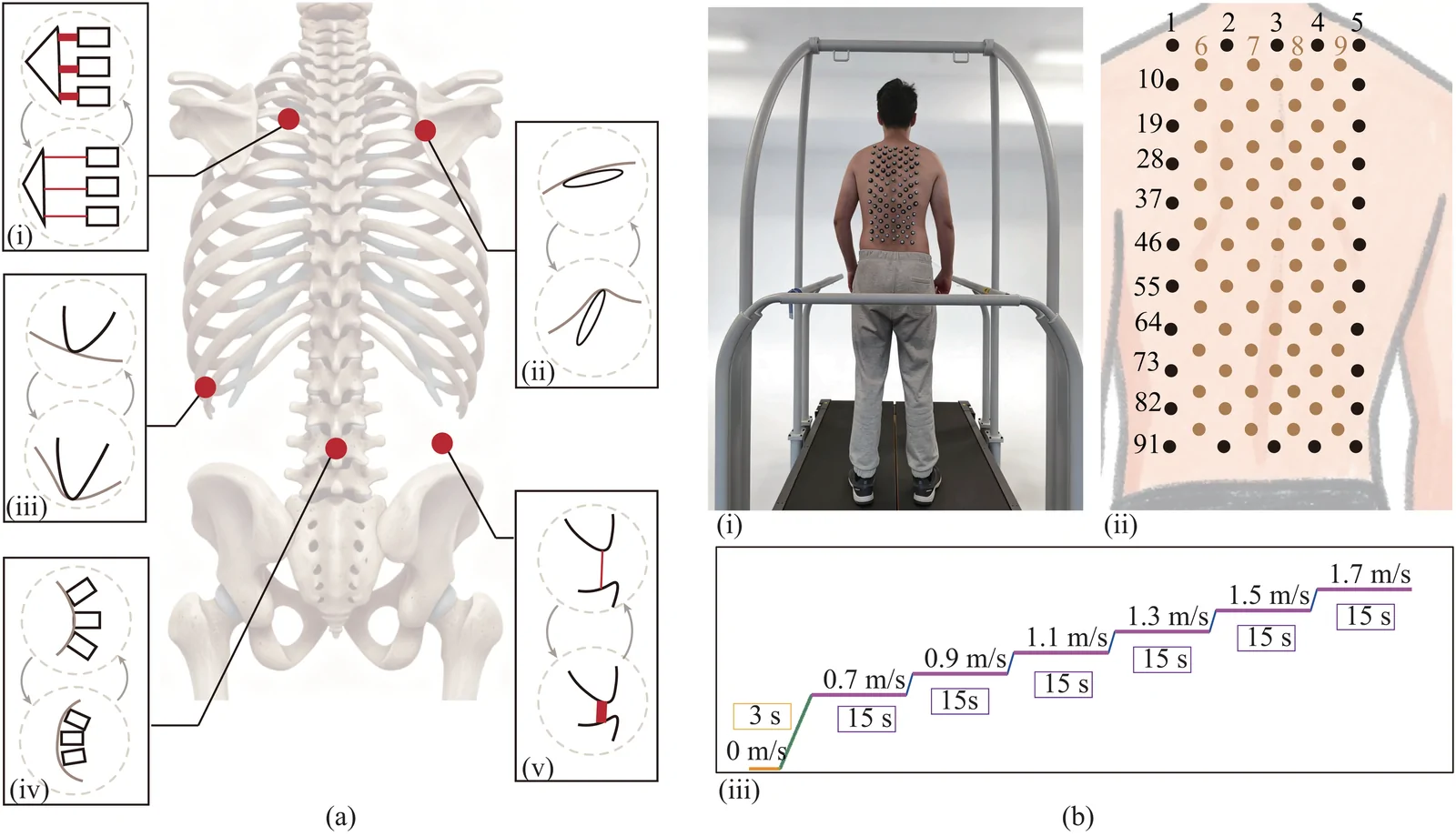
Illustration of typical dynamic surface topographical fluctuations and experimental setup. **(ai)** Changes in the relative position of the medial scapular border concerning the thoracic wall. **(aii)** Surface distortion due to scapular anterior tilting or winging. **(aiii)** Distinct surface deformations along the lower thoracic region caused by axial rotation and skin’s anisotropic properties. **(aiv)** Alteration of lumbar surface geometry due to lateral flexion and quadratus lumborum contraction. **(av)** Periodic elongation and shortening of the skin overlying the spinous processes due to lumbar flexion and extension during the gait cycle. **(bi)** Experimental scenario: Illustration of the overall testing environment. **(bii)** Marker configuration: Detailed placement of the 95 reflective markers distributed evenly across the subject’s dorsal surface. **(biii)** Speed protocol: The temporal profile of the walking speed on the treadmill, ranging from 0 m/s to 1.7 m/s, with each speed stage maintained for a duration of 15 seconds.

During dynamic functional activities such as walking, the dorsal body surface undergoes significant topographical fluctuations driven by the complex interplay between underlying skeletal kinematics and muscular dynamics [30]. In the upper back, the scapulothoracic rhythm necessitates cyclic alterations in the position of the medial scapular border relative to the thoracic wall (displayed in Fig 5ai). Movements such as scapular anterior tilting or winging (Fig 5aii) can significantly distort the overlying skin topography, while the concentric and eccentric contractions of the rhomboid major and minor muscles induce dynamic tension and stretching of the superficial integument [31]. Concurrently, axial rotation of the thoracic cage, combined with the anisotropic mechanical properties of the skin, results in distinct surface deformations along the lower thoracic region [32], as illustrated in Fig 5aiii. In the lumbar region, the lateral flexion inherent to the gait cycle requires contraction of the quadratus lumborum, which approximates the thoracic cage and the pelvis, thereby altering surface geometry (displayed in Fig 5aiv). Furthermore, the gait cycle involves rhythmic relative flexion and extension of the lumbar spine, causing periodic elongation and shortening of the skin overlying the spinous processes [33] (displayed in Fig 5av). Since these topographical alterations are intrinsic to and synchronized with the temporal phases of the gait cycle, quantitatively characterizing the dynamic morphology of the back surface is essential for understanding the biomechanical interface during locomotion.

### 5.3 Experimental protocol

A healthy male (age 28, height 187 cm, weight 83 kg) was recruited for the experiment. The experiment scenario was displayed in Fig 5bi. A total of 95 reflective markers were applied to the subject’s dorsal surface in a quasi-uniform grid pattern to capture skin movement. The detail of each marker set location was displayed in Fig 5bii. The subject was asked to walk on a force-sensing treadmill (Bertec FIT 5, Bertec, USA) under a series of controlled speed conditions. The walking protocol consisted of a ramp-up from 0.0 m/s to 1.7 m/s. Motion data were specifically collected at six distinct walking speeds: 0.7 m/s, 0.9 m/s, 1.1 m/s, 1.3 m/s, 1.5 m/s, and 1.7 m/s. Each speed condition was maintained for 15 seconds to ensure the capture of multiple stable gait cycles. The detailed speed change schedule is displayed in Fig 5biii. The time sequence of marker locations was continuously recorded across all walking speeds using the same optical motion capture system (Vicon Valkyrie 16, Oxford Metrics Limited, UK). Thus, there was no marker-specific acquisition delay among the marker trajectories. Concurrently, ground reaction force (GRF) data were recorded by the force plate sensors integrated within the treadmill. Marker trajectories were the primary data used for SDR network construction, whereas GRF data were used only for gait-event annotation. The marker location sequence signals were recorded at a sampling frequency of 100 Hz and subsequently subjected to low-pass filtering (Butterworth filter, 8 Hz cutoff frequency) to reduce the interference of irrelevant noise. All analytical procedures, including data pre-processing and functional segmentation, were implemented using customized MATLAB scripts (version 2021a, The MathWorks, Inc., Natick, USA).

### 5.4 Study flow

#### 5.4.1 Normalized neighborhood area.

**Fig 6:**
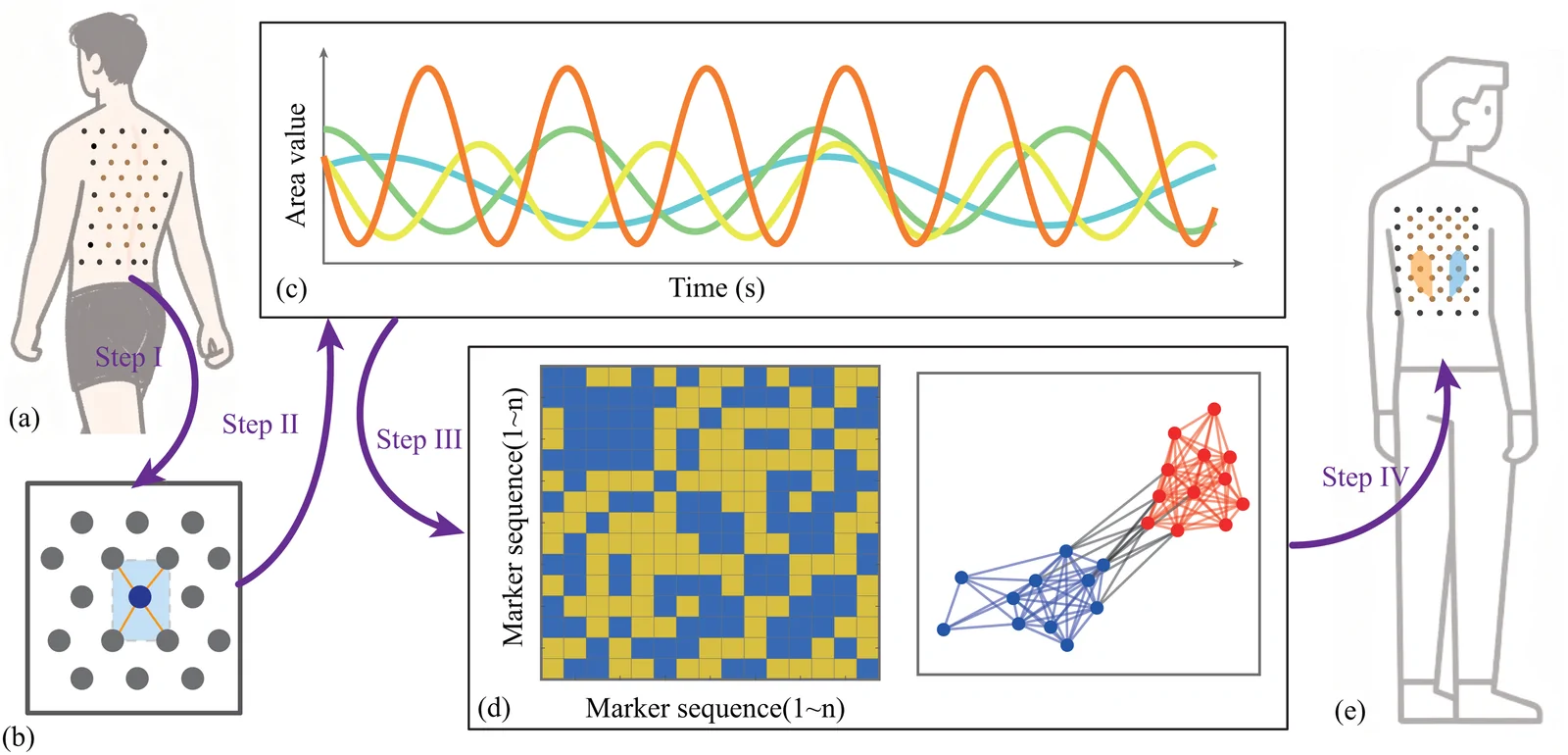
Computational workflow for functional segmentation of the dorsal skin. The diagram illustrates the step-by-step methodology used to transform raw marker position data into defined functional structural domains. **(a)** Marker application: Schematic showing the arrangement of the high-density reflective marker grid applied to the dorsal surface for motion capture. **(b)** Neighborhood area calculation: Detailed illustration of the method used to quantify local surface deformation. The neighborhood area of a central marker is defined by the combined area of four surrounding triangles, which is normalized by the static reference area to obtain the time-series sequence ${\bar{S}}_{i}(t)$. **(c)** Time-series area profile: Example of the normalized neighborhood area sequence (${\bar{S}}_{i}(t)$) over time, which serves as the fundamental input for the subsequent correlation analysis. **(d)** Network construction and Community Partition: Visualization combining the Spearman correlation matrix (heatmap) and the resulting thresholded correlation signed network. This step shows the application of community detection algorithms to group markers with strong synchronous ($\rho_{ij}>h$) and anti-phase ($\rho_{ij}<-h$) relationships. **(e)** Spatial mapping of functional domains: The final output showing the identified functional clusters mapped onto the anatomical dorsal surface, delineating areas of synchronized skin deformation behavior (structural domains).

The positions of markers are arranged in rows and columns, roughly forming a triangular lattice. To quantify the local surface deformation around each marker, we introduced the concept of a neighborhood area. The neighborhood area of a marker is defined as the combined area of the four triangles formed by connecting it to its four nearest neighbors (upper right, upper left, lower left, and lower right), as shown in Fig 6b. This area is calculated for each frame and normalized by its reference area obtained from the static posture


$$\begin{array}{c} {\bar{S}}_{i}(t)=\frac{S_{i}(t)}{S_{i}^{\text{static}}}, \end{array}$$

where *i* represents the marker number. It is important to note that markers on the periphery of the grid were used only to define the neighborhoods of the interior markers and were not included in the analysis.

#### 5.4.2 Spearman correlation matrix.

To investigate the structural characteristics of skin deformation on the back, we characterize the relationship between any two markers using the Spearman rank correlation coefficient. This non-parametric metric was calculated based on the rank-converted sequences of their normalized time-series neighborhood areas. The coefficient $\rho_{ij}$ between marker *i* and *j* is formally defined as


$$\begin{array}{c} \rho_{ij}=\frac{\text{cov}\left(\;R({\bar{S}}_{i}),R({\bar{S}}_{j})\right)}{\sigma_{R({\bar{S}}_{i})}\sigma_{R({\bar{S}}_{j})}}, \end{array}$$

where $R({\bar{S}}_{i})$ and $R({\bar{S}}_{j})$ represent the rank variables of the normalized time-series area sequences ${\bar{S}}_{i}$ and ${\bar{S}}_{j}$, respectively. The coefficients for all marker pairs were subsequently compiled into the Spearman correlation matrix, $C=(\rho_{ij}{)}_{n\times n}$.

#### 5.4.3 Correlation signed network.

We constructed a marker correlation signed network (𝒢), where the nodes represent the individual markers. The links in the network correspond to the pairwise correlations, with their weights derived from the correlation matrix *C*. To ensure that the functional structure reflects only strong and coordinated activity during the walking cycle, we implemented a strict filtering process. We retained links with an absolute correlation coefficient greater than *h* = 0.8. The weighted adjacency matrix $A=(a_{ij}{)}_{\left(n\times n\right)}$ of the network is thus defined as


$$\begin{array}{c} a_{ij}=\left\{ \begin{array}{cc} \rho_{ij}, & \text{if }|\rho_{ij}|>h\\ 0, & \text{otherwise} \end{array}\right. \end{array}$$ (1)

This filtering process results in the final correlation signed network, denoted by 𝒢, where only strong kinematic coupling is preserved.

#### 5.4.4 Grouping synchronous markers.

To identify groups of markers that exhibit synchronous stretching or contraction patterns, we performed community detection on the signed correlation network 𝒢. The objective is to find a partition where markers within the same group are more strongly positively correlated with each other than with markers in other groups. The procedure consists of the following two steps.

First, we applied modularity-based community detection to the unsigned version of the correlation network (i.e., using the absolute values of the correlations). Modularity quantifies the quality of a network partition by measuring the density of connections within clusters compared to a random null model [34]. We used the Louvain algorithm [35] as implemented in Gephi [36] to find the partition that maximizes modularity. To ensure robustness, we performed 10 independent runs for each network and selected the partition with the highest modularity value.

Second, within each community identified from the unsigned network, we further accounted for the sign structure of the correlations. Specifically, we examined whether the subgraph induced by each community was structurally balanced—that is, whether it could be partitioned into two subgroups such that all positive edges lie within subgroups and all negative edges lie between them. If such a bipartition existed, we split the original community accordingly; otherwise, we applied the signed Louvain algorithm to obtain the bipartition. In the context of the present study, all identified communities were found to be bipartite, leading to a clear binary partition of the network.

Additional robustness analyses were performed to evaluate the sensitivity of SDR identification to network threshold selection, marker-density reduction, and temporal windowing. These analyses repeated the SDR-identification pipeline under alternative correlation thresholds, lower density marker layouts, and 5-s moving windows. Full procedures and results are provided in S1 Text.

## Supporting information

S1 Text Robustness and sensitivity analysis of SDR identification. This supporting text provides detailed methods and results for the three robustness analyses added in the revised manuscript: threshold-sensitivity analysis, marker-density sensitivity analysis, and moving-window temporal-stability analysis. It also includes embedded supplementary figures and a compact summary table for the threshold-sensitivity analysis.
(PDF)

## References

[1] Ferreira ML, de Luca K, Haile LM, Steinmetz JD, Culbreth GT, Cross M, et al. Global, regional, and national burden of low back pain, 1990–2020, its attributable risk factors, and projections to 2050: a systematic analysis of the Global Burden of Disease Study 2021. Lancet Rheumatol. 2023;5(6):e316-29. doi: 10.1016/S2665-9913(23)00098-X PMC10234592 37273833

[2] Force UPST. Screening for adolescent idiopathic scoliosis: evidence report and systematic review for the US preventive services task force. JAMA. 2018;319(2):165–72. doi: 10.1001/jama.2017.19342 29318283 PMC13480065

[3] Cohen SP, Argoff CE, Carragee EJ. Management of low back pain. BMJ. 2008;337:a2718. doi: 10.1136/bmj.a2718 19103627

[4] Michelini G, Corridore A, Torlone S, Bruno F, Marsecano C, Capasso R, et al. Dynamic MRI in the evaluation of the spine: state of the art. Acta Biomed. 2018;89(1-S):89–101. doi: 10.23750/abm.v89i1-S.7012 29350639 PMC6179074

[5] Duncombe P, Dick T, Ng PTT, Izatt MT, Labrom RD, Tucker K. Beyond the Curve: The Muscle-Specific Asymmetries of Adolescent Idiopathic Scoliosis. JOR Spine. 2025;8(4):e70129. doi: 10.1002/jsp2.70129 41194969 PMC12585792

[6] Cheung WK, Cheung JPY, Lee W-N. Role of Ultrasound in Low Back Pain: A Review. Ultrasound Med Biol. 2020;46(6):1344–58. doi: 10.1016/j.ultrasmedbio.2020.02.004 32192782

[7] Park J-H, Stegall PR, Roye DP, Agrawal SK. Robotic Spine Exoskeleton (RoSE): Characterizing the 3-D Stiffness of the Human Torso in the Treatment of Spine Deformity. IEEE Trans Neural Syst Rehabil Eng. 2018;26(5):1026–35. doi: 10.1109/TNSRE.2018.2821652 29752238

[8] Murray RC, Ophaswongse C, Park J-H, Agrawal SK. Characterizing Torso Stiffness in Female Adolescents With and Without Scoliosis. IEEE Robot Autom Lett. 2020;5(2):1634–41. doi: 10.1109/lra.2020.2969945

[9] Lerchl T, Nispel K, Bodden J, Sekuboyina A, El Husseini M, Fritzsche C, et al. Musculoskeletal spine modeling in large patient cohorts: how morphological individualization affects lumbar load estimation. Front Bioeng Biotechnol. 2024;12:1363081. doi: 10.3389/fbioe.2024.1363081 38933541 PMC11199547

[10] Ignasiak D, Ferguson SJ, Arjmand N. A rigid thorax assumption affects model loading predictions at the upper but not lower lumbar levels. J Biomech. 2016;49(13):3074–8. doi: 10.1016/j.jbiomech.2016.07.006 27515441

[11] Schmid S, Connolly L, Moschini G, Meier ML, Senteler M. Skin marker-based subject-specific spinal alignment modeling: A feasibility study. J Biomech. 2022;137:111102. doi: 10.1016/j.jbiomech.2022.111102 35489234

[12] Betsch M, Wild M, Johnstone B, Jungbluth P, Hakimi M, Kühlmann B, et al. Evaluation of a novel spine and surface topography system for dynamic spinal curvature analysis during gait. PLoS One. 2013;8(7):e70581. doi: 10.1371/journal.pone.0070581 23894674 PMC3720906

[13] Michalik R, Knod M, Siebers H, Gatz M, Dirrichs T, Eschweiler J, et al. Introduction and evaluation of a novel multi-camera surface topography system. Gait Posture. 2020;80:367–73. doi: 10.1016/j.gaitpost.2020.06.016 32619923

[14] Jang D, Park S. A morphometric study of the lumbar interspinous space in 100 stanford university medical center patients. J Korean Neurosurg Soc. 2014;55(5):261–6. doi: 10.3340/jkns.2014.55.5.261 25132932 PMC4130951

[15] van Amstel RN, Weide G, Wesselink EO, Noten K, Jacobs K, Pool-Goudzwaard AL, et al. A review and empirical findings of fasciae and muscle interactions in low back pain. Front Physiol. 2025;16:1604459. doi: 10.3389/fphys.2025.1604459 41000111 PMC12457458

[16] Sarıoğlu K, Bayrakcı Tunay V. Influence of thoracolumbar mobility on running performance: A comparative study. Appl Sci. 2025;15(5):2777. doi: 10.3390/app15052777

[17] Procópio PRS, Pinto RZ, Murta BAJ, Caldeira PF, Araújo PA, Schleip R, et al. Individuals with chronic low back pain have reduced myofascial force transmission between latissimus dorsi and contralateral gluteus maximus muscles. J Biomech. 2025;190:112850. doi: 10.1016/j.jbiomech.2025.112850 40616971

[18] Myers TW. Anatomy trains: myofascial meridians for manual and movement therapists. Edinburgh: Churchill Livingstone Elsevier; 2014.

[19] Gimmon Y, Riemer R, Rashed H, Shapiro A, Debi R, Kurz I, et al. Age-related differences in pelvic and trunk motion and gait adaptability at different walking speeds. J Electromyogr Kinesiol. 2015;25(5):791–9. doi: 10.1016/j.jelekin.2015.05.003 26091623

[20] Ní Annaidh A, Bruyère K, Destrade M, Gilchrist MD, Otténio M. Characterization of the anisotropic mechanical properties of excised human skin. J Mech Behav Biomed. 2012;5(1):139–48. doi: 10.1016/j.jmbbm.2011.08.016 22100088

[21] Hussain SH, Limthongkul B, Humphreys TR. The biomechanical properties of the skin. Dermatol Surg. 2013;39(2):193–203. doi: 10.1111/dsu.12095 23350638

[22] Humphrey JD. Continuum biomechanics of soft biological tissues. Proc A. 2003;459(2029):3–46. doi: 10.1098/rspa.2002.1060

[23] Sherman VR, Tang Y, Zhao S, Yang W, Meyers MA. Structural characterization and viscoelastic constitutive modeling of skin. Acta Biomater. 2017;53:460–9. doi: 10.1016/j.actbio.2017.02.011 28219806

[24] Milosevic M, Yokoyama H, Grangeon M, Masani K, Popovic MR, Nakazawa K, et al. Muscle synergies reveal impaired trunk muscle coordination strategies in individuals with thoracic spinal cord injury. J Electromyogr Kinesiol. 2017;36:40–8. doi: 10.1016/j.jelekin.2017.06.007 28734171

[25] Saito H, Yokoyama H, Sasaki A, Nakazawa K. Muscle synergy patterns as altered coordination strategies in individuals with chronic low back pain: a cross-sectional study. J Neuroeng Rehabil. 2023;20(1):69. doi: 10.1186/s12984-023-01190-z 37259142 PMC10230697

[26] Saito H, Yokoyama H, Sasaki A, Matsushita K, Nakazawa K. Variability of trunk muscle synergies underlying the multidirectional movements and stability trunk motor tasks in healthy individuals. Sci Rep. 2023;13(1):1193. doi: 10.1038/s41598-023-28467-6 36681745 PMC9867711

[27] Malakoutian M, Sanchez CA, Brown SHM, Street J, Fels S, Oxland TR. Biomechanical Properties of Paraspinal Muscles Influence Spinal Loading-A Musculoskeletal Simulation Study. Front Bioeng Biotechnol. 2022;10:852201. doi: 10.3389/fbioe.2022.852201 35721854 PMC9201424

[28] Chwała W, Koziana A, Kasperczyk T, Walaszek R, Płaszewski M. Electromyographic assessment of functional symmetry of paraspinal muscles during static exercises in adolescents with idiopathic scoliosis. Biomed Res Int. 2014;2014:573276. doi: 10.1155/2014/573276 25258713 PMC4167233

[29] Hodges PW, Tucker K. Moving differently in pain: a new theory to explain the adaptation to pain. Pain. 2011;152(3 Suppl):S90–8. doi: 10.1016/j.pain.2010.10.020 21087823

[30] Zemp R, List R, Gülay T, Elsig JP, Naxera J, Taylor WR, et al. Soft tissue artefacts of the human back: comparison of the sagittal curvature of the spine measured using skin markers and an open upright MRI. PLoS One. 2014;9(4):e95426. doi: 10.1371/journal.pone.0095426 24748013 PMC3991691

[31] Ludewig PM, Cook TM. Alterations in shoulder kinematics and associated muscle activity in people with symptoms of shoulder impingement. Phys Ther. 2000;80(3):276–91. doi: 10.1093/ptj/80.3.276 10696154

[32] Maiti R, Gerhardt L-C, Lee ZS, Byers RA, Woods D, Sanz-Herrera JA, et al. In vivo measurement of skin surface strain and sub-surface layer deformation induced by natural tissue stretching. J Mech Behav Biomed Mater. 2016;62:556–69. doi: 10.1016/j.jmbbm.2016.05.035 27310571

[33] Feipel V, De Mesmaeker T, Klein P, Rooze M. Three-dimensional kinematics of the lumbar spine during treadmill walking at different speeds. Eur Spine J. 2001;10(1):16–22. doi: 10.1007/s005860000199 11276830 PMC3611469

[34] Newman MEJ, Girvan M. Finding and evaluating community structure in networks. Phys Rev E. 2004;69(2 Pt 2):026113. doi: 10.1103/PhysRevE.69.026113 14995526

[35] Blondel VD, Guillaume J-L, Lambiotte R, Lefebvre E. Fast unfolding of communities in large networks. J Stat Mech. 2008;2008(10):P10008. doi: 10.1088/1742-5468/2008/10/p10008

[36] Bastian M, Heymann S, Jacomy M. Gephi: An Open Source Software for Exploring and Manipulating Networks. ICWSM. 2009;3(1):361–2. doi: 10.1609/icwsm.v3i1.13937

