# Supplementary material for: Network analysis of surface deformation reveals trunk modularity and synchronization during gait
Source: PLoS Comput Biol. 2026 Jul 24;22(7):e1014388. doi: 10.1371/journal.pcbi.1014388 (PMC13524338; doi:10.1371/journal.pcbi.1014388)
Supplement: Supplementary file 1 [file pcbi.1014388.s001.pdf]

Supporting Information: Robustness and Sensitivity Analysis of SDR Identification

Zilu Wang[1,#], Jingbang Yang[1,#], Yong Wang[1], Tiantong Wang[3], Heran Zhong[1], Fengchen Liu[4,5], Chenxi Zhang[1], Jiangtian Li[1], Rongli Wang[2], Ximing Xu[6], Jiangang Shi[6], Sunil K. Agrawal[7,8], Qining Wang[1,2,4,*]

1 School of Advanced Manufacturing and Robotics, Peking University, Beijing 100871, China

2 Department of Rehabilitation Medicine, First Hospital, Peking University, Beijing 100034, China

3 Key Laboratory of Biomimetic Robots and Systems of Ministry of Education, School of Mechatronical Engineering, Beijing Institute of Technology, Beijing 100081, China

4 School of Rehabilitation Sciences and Engineering, University of Health and Rehabilitation Sciences, Qingdao 266071, China

5 School of Biological Science and Medical Engineering, Beihang University, Beijing 100191, China

6 Department of Orthopedic Surgery, Changzheng Hospital, Shanghai 200003, China

7 Robotics and Rehabilitation Laboratory, Department of Mechanical Engineering, Columbia University, New York, NY 10027, USA

8 Department of Rehabilitation and Regenerative Medicine, Columbia University Medical Center, New York, NY 10027, USA

[#] These authors contributed equally to this work.

[*] qiningwang@pku.edu.cn

To ensure that the identified synchronous deformation regions (SDRs) represent physiological coordination rather than methodological artifacts, we conducted sensitivity analyses on three aspects of the computational pipeline: hyperparameter selection (threshold h), spatial resolution, and temporal stationarity. The supporting information text is organized as follows: In Section A, we evaluate the topological stability of the SDRs across different network sparsity levels induced by varying the correlation threshold h. In Section B, we determine whether the identified SDRs are recoverable when the dorsal surface is sampled at reduced spatial resolutions. In Section C, we assess the temporal continuity of the SDR partitions to confirm that the full-segment organization is not distorted by localized dynamic shifts between the beginning and end of the walking trial.

A. Threshold-sensitivity analysis

In the main analysis, we constructed a signed correlation network by retaining strong pairwise Spearman correlations between markers, using an absolute correlation threshold of $h = 0.80$. Because strict threshold can alter graph sparsity, connectedness, modularity, and the resulting community partition, we performed an explicit threshold-sensitivity analysis to determine whether the identified synchronous deformation regions (SDRs) were dependent on this hyperparameter.

A.1. Methods

The complete SDR-identification pipeline was repeated for five correlation thresholds:

$$h = 0.70, 0.75, 0.80, 0.85, 0.90.$$

For each walking speed (0.7, 0.9, 1.1, 1.3, 1.5, and 1.7 m/s), local normalized

neighborhood area time series were computed from the dorsal marker trajectories using the same preprocessing procedure as in the main analysis. Spearman correlation coefficients were then computed for all pairs of analyzed inner markers. For a given threshold h, the signed weighted adjacency matrix was defined as

$$a_{ij}^{(h)} = \begin{cases} \rho_{ij}, & |\rho_{ij}| > h, \\ 0, & \text{otherwise,} \end{cases}$$

where ρ_{ij} is the Spearman correlation coefficient between normalized neighborhood area time series at markers i and j. The diagonal entries were set to zero. The same statistical filtering and SDR-detection procedure used in the main analysis was applied at each threshold. Briefly, modularity-based community detection was performed on the unsigned version of the network, and the sign structure of the corresponding signed network was then used to subdivide communities into positively connected SDRs.

The partition obtained at $h = 0.80$ was used as the reference because this was the threshold used in the main manuscript. Partitions obtained at the other thresholds were compared with the $h = 0.80$ reference partition using adjusted Rand index (ARI), normalized mutual information (NMI), and Jaccard overlap of matched major SDRs. For each speed, the five largest SDRs in the $h = 0.80$ reference partition were treated as major reference SDRs. Each reference SDR was matched to the SDR at another threshold that maximized Jaccard overlap. To quantify spatial boundary shifts, we also computed the centroid displacement and relative area change of the matched SDRs. Network-level effects of thresholding were assessed using edge count, edge density, positive and negative edge counts, number of connected components, largest connected-component fraction, modularity Q, number of Louvain communities, and

number of final SDRs.

To facilitate visual comparison, the SDR partition at each walking speed was used as the reference color template. Major SDRs preserved across partitions were mapped to a fixed color palette: blue for the left middle major SDR, red for the right middle major SDR, grey for the spinal/midline SDR, tan for the lower-left SDR, and light blue for the lower-right SDR. Singleton or very small communities are shown in black. Note that this color coding was applied only for illustration purposes and did not alter the underlying SDR labels, partition-similarity metrics, or spatial-overlap calculations.

A.2. Results

Changing the threshold altered network sparsity in the expected direction. The mean edge density across walking speeds decreased monotonically from 0.278 at $h = 0.70$ to 0.188 at $h = 0.80$ and 0.078 at $h = 0.90$. The mean modularity Q increased from 0.260 at $h = 0.70$ to 0.338 at $h = 0.80$ and 0.567 at $h = 0.90$, consistent with the progressive pruning of weaker edges and increased graph fragmentation. These network-level metrics are summarized in Table A and Fig A in S1 Text.

Table A. Compact summary of threshold-sensitivity metrics.

h	Edge density	Q	ARI	NMI	Jaccard	Max Δc (mm)
**0.70**	0.278	0.260	0.776	0.839	0.776	285.3
**0.75**	0.237	0.286	0.828	0.888	0.828	204.5
**0.80**	0.188	0.338	1.000	1.000	1.000	0.0
**0.85**	0.130	0.450	0.898	0.931	0.854	261.4

**0.90**	0.078	0.567	0.700	0.822	0.649	261.4

Values are summarized across walking speeds. Edge density, modularity Q, ARI, NMI, and major-SDR Jaccard overlap are reported as across-speed means. Max centroid shift Δc denotes the maximum centroid displacement observed among the matched major SDRs.

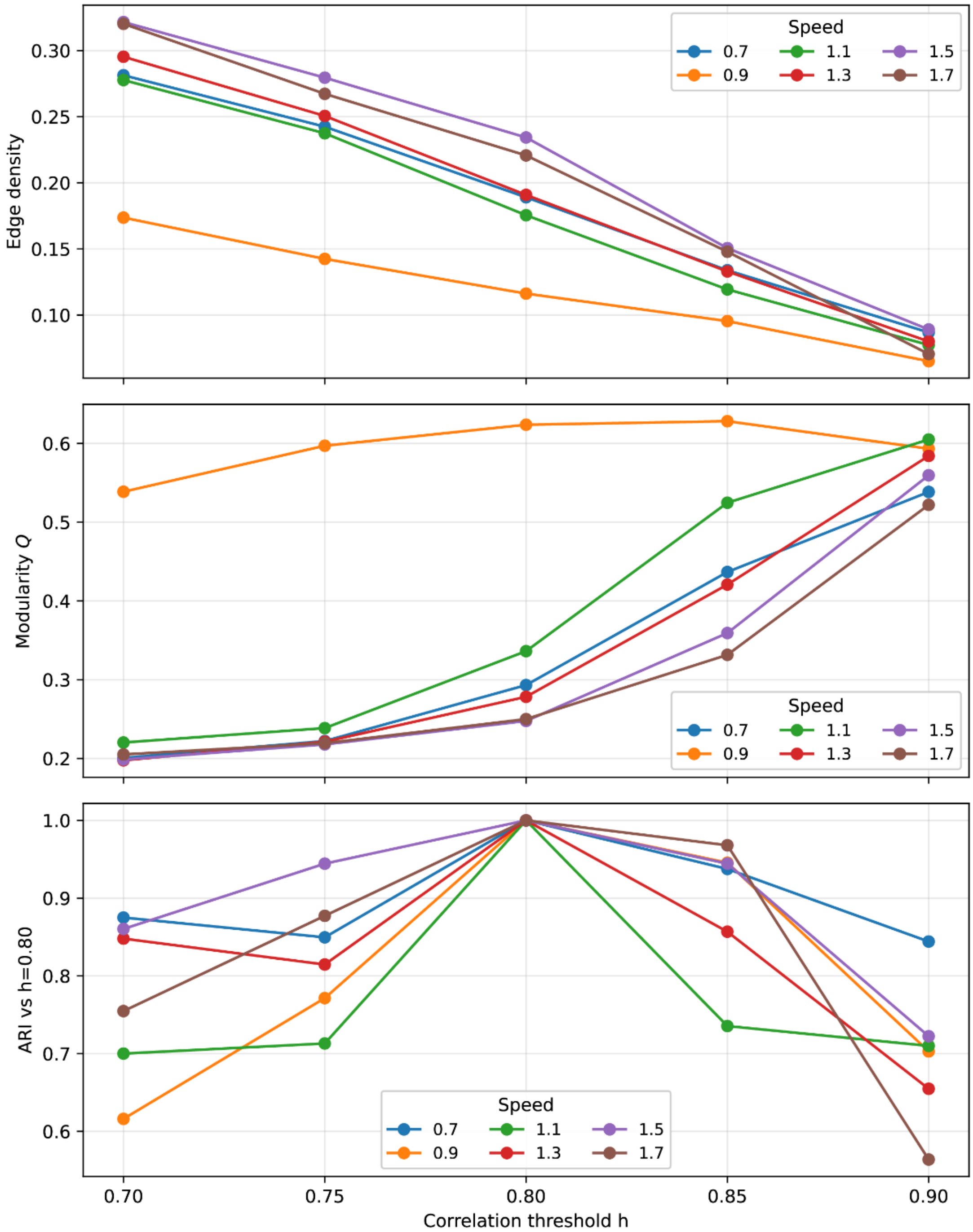

Fig A. Network-level and partition-level threshold-sensitivity metrics. Edge density decreased monotonically with increasing threshold, whereas modularity Q increased because stronger thresholding produced sparser and more fragmented graphs. ARI values were computed relative to the $h = 0.80$ reference partition at the same walking speed. The partition

similarity remained high for moderate threshold perturbations, particularly at $h = 0.75$ and $h = 0.85$, while highest threshold $h = 0.90$ produced larger changes in small peripheral clusters.

Despite these changes in graph density, the principal SDR organization remained largely stable relative to the $h = 0.80$ reference partition. Across all non-reference thresholds, ARI ranged from 0.564 to 0.968 and NMI ranged from 0.696 to 0.969. For the moderate threshold perturbations $h = 0.75$ and $h = 0.85$, the mean ARI was 0.863 and the mean NMI was 0.910, indicating high partition similarity to the reference solution. The mean Jaccard overlap of the five major SDRs was 0.841 across $h = 0.75$ and $h = 0.85$.

The two dominant lateral SDRs were especially stable. Across non-reference thresholds, the two largest SDRs showed mean Jaccard overlaps of 0.866, 0.886, 0.939, and 0.818 at $h = 0.70$, $h = 0.75$, $h = 0.85$, and $h = 0.90$, respectively. Their mean centroid displacements were 9.7 mm, 11.9 mm, 5.8 mm, and 18.3 mm at these thresholds, respectively. These values indicate that the core left-right thoracolumbar SDRs were not created by the specific choice of $h = 0.80$.

At the largest threshold, $h = 0.90$, weaker long-range and peripheral edges were pruned more aggressively. This reduced mean ARI to 0.700, mean NMI to 0.822, and mean major-SDR Jaccard overlap to 0.649. The spatial maps show that the main bilateral and midline organization remained identifiable, whereas small peripheral SDRs and singleton or tiny communities were more threshold-sensitive.

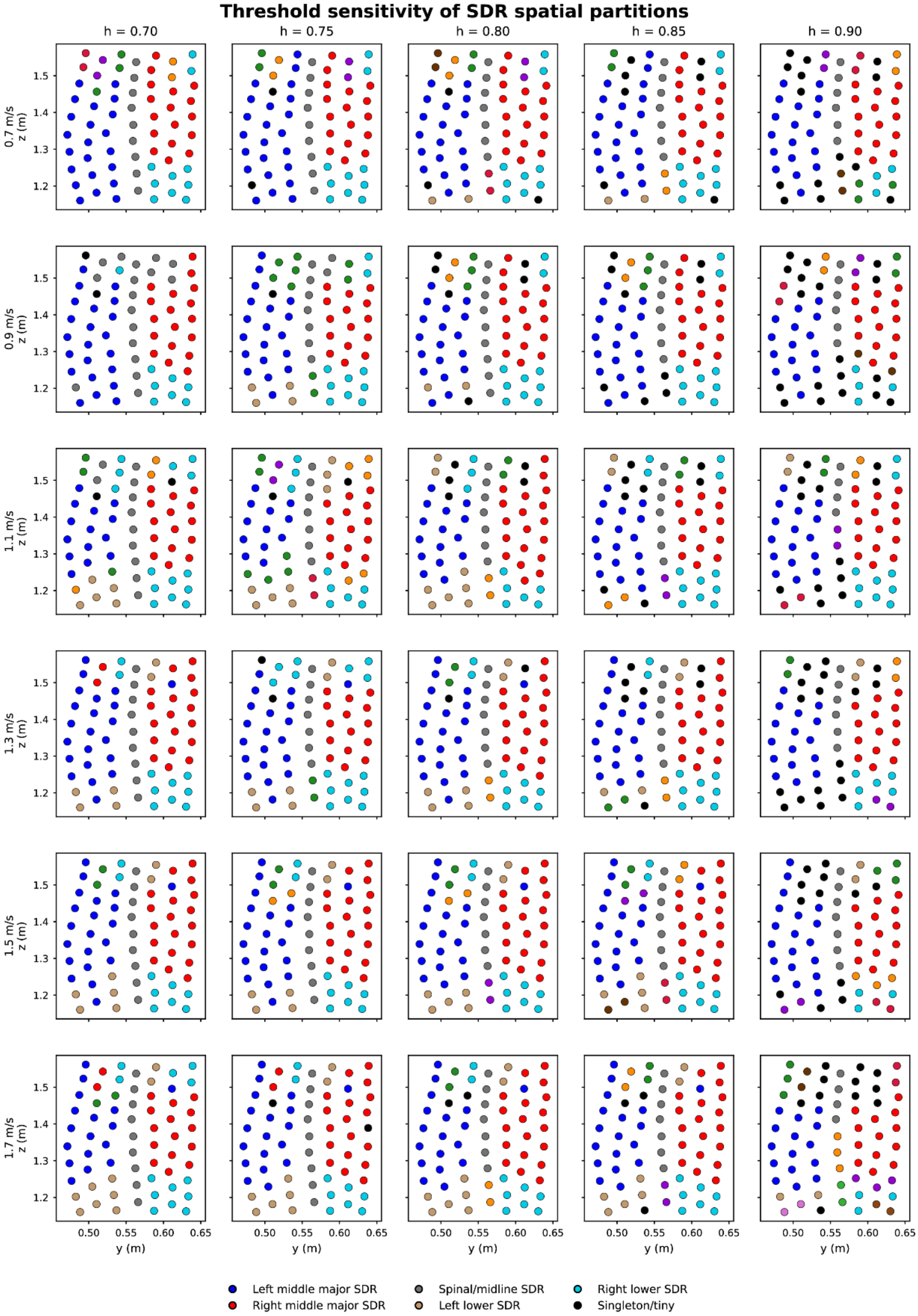

Fig B. Threshold sensitivity of SDR spatial partitions. SDR partitions were reconstructed using correlation thresholds $h = 0.70$, $h = 0.75$, $h = 0.80$, $h = 0.85$ and $h = 0.90$ for all six walking speeds. Each panel shows the $y - z$ projection of the analyzed inner markers. For visualization, the $h = 0.80$ partition at each walking speed was used as the reference colour template, and SDRs detected at other thresholds were matched to the reference by maximum Jaccard overlap. Major SDRs preserved across partitions were mapped to a fixed

color palette: blue for the left middle major SDR, red for the right middle major SDR, grey for the spinal/midline SDR, tan for the lower-left SDR, and light blue for the lower-right SDR. Singleton or very small communities are shown in black. The principal bilateral and midline SDRs remained identifiable across the tested thresholds, whereas smaller peripheral SDRs were more threshold-sensitive.

B. Marker-density sensitivity

To evaluate whether SDR identification depended on the dense 95-marker configuration, we performed a marker-density sensitivity analysis using two regular reduced layouts.

B.1. Methods

The original marker field formed a staggered nine-column layout in the dorsal $y-z$ projection. Removing markers may disrupt the original four-triangle local neighborhood structure used in the full-density analysis. Strictly enforcing this four-triangle structure would have necessitated the removal of a substantial number of additional nodes, resulting in an overly sparse topology. Consequently, this approach was not pursued. Instead, we generated two complementary, regular, reduced-density layouts by retaining either odd or even physical marker columns. Columns were defined by sorting the nine vertical marker columns according to their mean static y-coordinates, and used consistently across all walking speeds. Specifically, the odd-column layout retained physical columns 1, 3, 5, 7, and 9, whereas the even-column layout retained columns 2, 4, 6, and 8. The odd-column layout retained 55 markers. Among these retained markers, 27 were original inner markers used as network nodes, and 28 were original boundary markers used only for local surface-area calculation. The even-column layout retained the 40 even-column markers as network nodes and additionally

retained the original 28 boundary markers as geometric support points for neighborhood area calculation. This correction is necessary because some even-column markers that are inner markers in the full-density grid become boundary-like points after the odd columns are removed. We therefore retained the original boundary markers in the even-column layout to preserve valid neighborhood area definitions and the spatial confines of the measurement domain. Thus, the even-column layout contained 68 retained markers in total, of which 40 were analyzed as network nodes.

Since removing complete marker columns breaks the original full-density neighborhood structure, the reduced layouts required new but fixed local neighborhood definitions. We defined layout-specific neighborhoods based on the regular marker arrangement, following the same principle as the main analysis: the neighborhood area of a marker was computed as the sum of the areas of triangles formed by the marker and its surrounding support points.

For the odd-column layout, the retained markers formed an approximately rectangular lattice. For each analyzed marker, the local neighborhood was defined by its four surrounding retained points in the up, right, down, and left directions. The local area was then computed as the sum of the four triangle areas formed by the center marker and each adjacent pair of surrounding markers.

For the even-column layout, the retained set consisted of the even-column analyzed markers and the original boundary markers. For each analyzed marker, a fixed support-assisted ring of surrounding retained markers was defined. The local area was computed

as the sum of the areas of the triangles formed by the center marker and each pair of adjacent markers in this ring. For example, the neighborhood ring of Marker 6 was defined using Markers 1, 2, 7, 10, and 15; the neighborhood ring of Marker 16 was defined using Markers 7, 15, 17, and 25. The fixed neighborhood topology used for the reduced layouts is illustrated in Fig C in S1 Text.

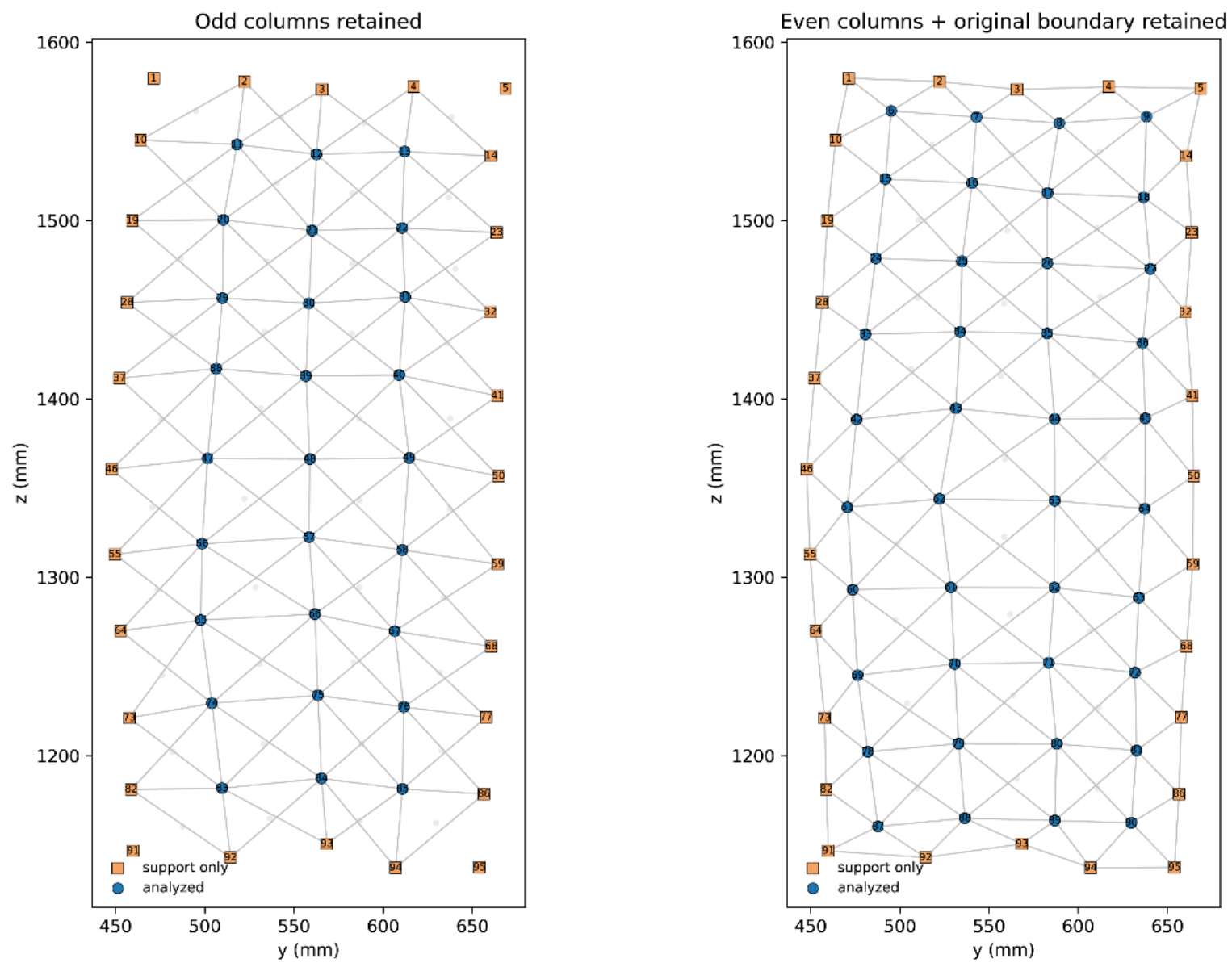

Fig C. Fixed neighborhood topology used in the marker-density sensitivity analysis. The odd-column and even-column layouts were assigned fixed local neighborhood topologies based on the static marker lattice. Blue circles indicate analyzed markers used as network nodes, orange squares indicate boundary markers used only for local surface-area calculation, and grey lines indicate the local triangular neighborhoods used to compute reduced-density local area. The same neighborhood topology was used for all walking speeds.

For each walking speed, the same network-analysis pipeline was then applied to the reduced-density neighborhood area time series: Spearman correlation calculation, FDR filtering, thresholding at $h = 0.80$, modularity-based community detection on the unsigned network, and sign-based subdivision into positively connected SDRs. The reduced-density partitions were compared with the full-density $h = 0.80$ reference

partition on the retained markers. We quantified adjusted Rand index (ARI), normalized mutual information (NMI), Jaccard overlap of the five major full-density SDRs, centroid displacement of matched SDRs, edge density, modularity Q, and the number of detected SDRs.

B.2. Results

Both reduced layouts recovered part of the full-density SDR organization, but neither layout fully preserved the detailed full-density partition. Across walking speeds, the odd-column layout yielded mean ARI = 0.550, mean NMI = 0.761, mean major-SDR Jaccard overlap = 0.654, and mean centroid displacement = 40.3 mm. The even-column layout with original boundary markers yielded mean ARI = 0.594, mean NMI = 0.675, mean major-SDR Jaccard overlap = 0.628, and mean centroid displacement = 57.2 mm. This indicates that the two regular reduced layouts can preserve some coarse bilateral deformation organization, but they do not reliably reproduce the full-density SDR boundaries.

These results support the use of the original 95-marker configuration for the present high-resolution proof-of-concept analysis. The dense marker layout was not simply redundant; it provided the spatial support required to define local surface neighborhoods and to resolve smaller or boundary-sensitive SDRs. Future simplified clinical marker sets may be feasible, but they should be optimized and validated separately rather than assumed from simple column removal.

The spatial comparison is shown in Fig D in S1 Text. The corresponding quantitative similarity metrics, including ARI, NMI, major-SDR Jaccard overlap, and centroid displacement, are shown in Fig E in S1 Text.

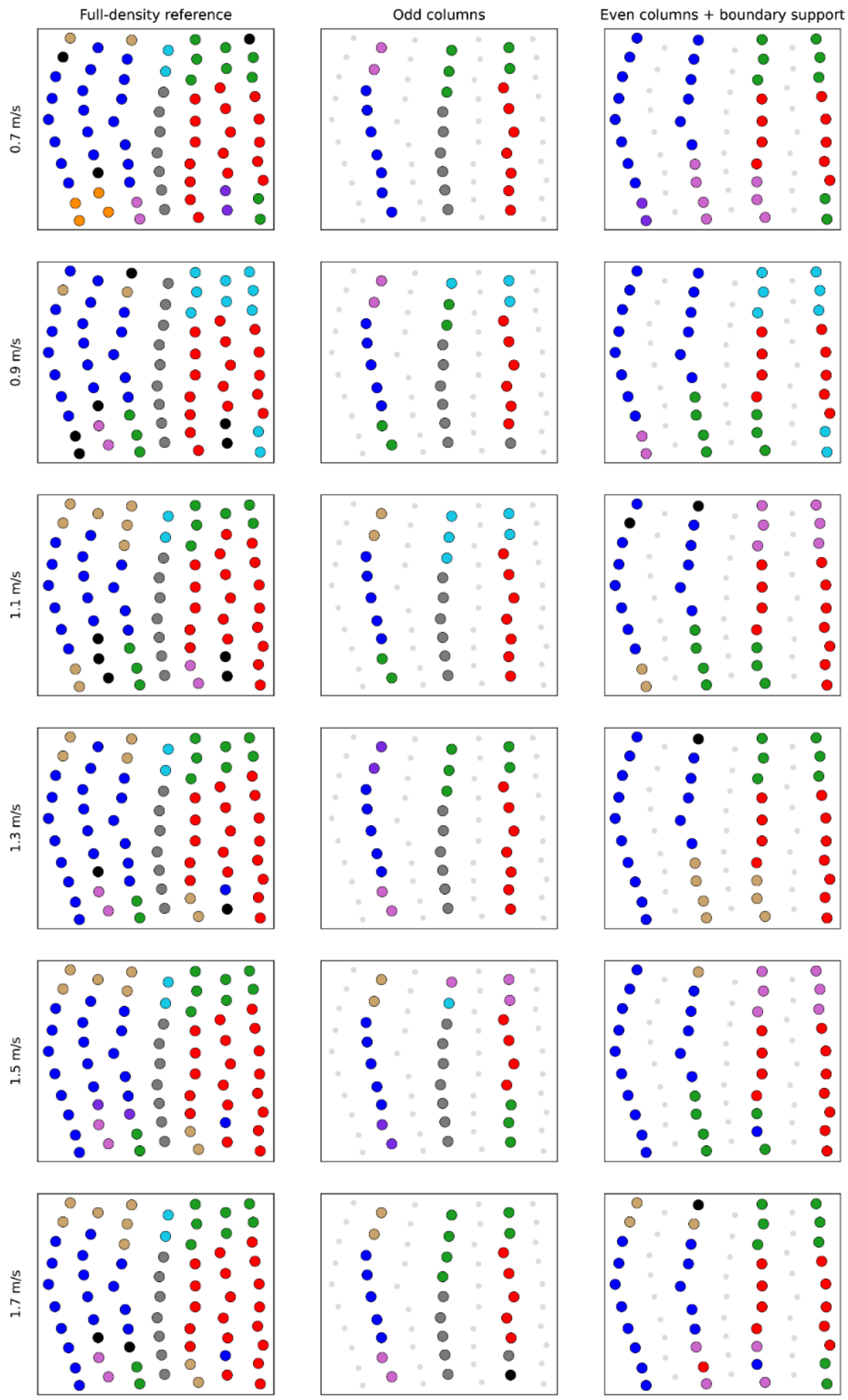

Fig D. Column-layout marker-density sensitivity of SDR spatial partitions. Full-density $h = 0.80$ SDR partitions are compared with two regular reduced-density layouts. The odd-column layout retained 55 markers, of which 27 were analyzed as network nodes and 28 were

used as boundary markers. The even-column layout retained 40 analyzed markers together with the original 28 boundary markers, yielding 68 retained markers in total. In the reduced-layout panels, light-grey dots indicate omitted full-density inner markers, whereas colored dots indicate retained analyzed markers. The reduced layouts recover some coarse bilateral organization but do not reproduce the detailed full-density SDR boundaries.

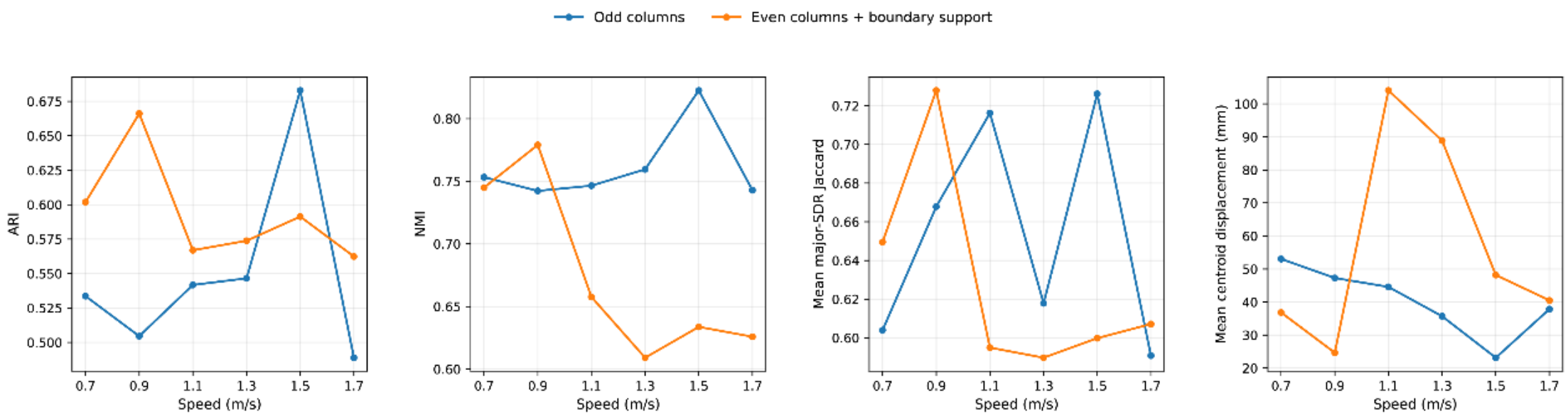

Fig E. Column-layout marker-density sensitivity metrics. ARI, NMI, mean major-SDR Jaccard overlap, and mean centroid displacement were computed by comparing the reduced-density SDR partitions with the full-density $h = 0.80$ reference partition on the retained analyzed markers. Both the odd-column and even-column reduced layouts showed partial recovery of the full-density SDR structure, but neither fully reproduced the detailed full-density partition.

C. Moving-window temporal-stability analysis

To examine whether the SDR structure changes between the beginning and the end of walking, we performed a moving-window temporal-stability analysis. The purpose was to evaluate whether the full-segment SDR partition represents a stable principal deformation organization or whether substantially different SDR partitions emerge at different times within the walking segment.

C.1. Methods

For each walking speed, we used the same selected walking segment as in the main analysis. Local normalized neighborhood area time series were computed from the marker trajectories using the same preprocessing, filtering, and neighborhood area

calculation procedure as in the main analysis. The selected walking segment was then divided into 5-s windows with a 1-s step.

For each 5-s window, the complete SDR-identification pipeline was repeated: Spearman correlation calculation, FDR filtering, construction of the signed network at $h = 0.80$, modularity-based community detection on the unsigned network, and sign-based subdivision into positively connected SDRs. The full-segment $h = 0.80$ SDR partition at the corresponding walking speed was used as the reference partition. Window-level partitions were compared with the full-segment reference using adjusted Rand index (ARI), normalized mutual information (NMI), Jaccard overlap of the five major full-segment SDRs, and centroid displacement of matched major SDRs. We also computed network-level metrics including edge density, modularity Q, and the number of detected SDRs.

To assess temporal continuity, adjacent-window ARI and NMI were computed between consecutive 5-s windows. To directly address the reviewer's concern regarding the beginning and end of walking, we also compared the first 5-s window with the final 5-s window of the same selected walking segment. Because the original frame selection used in the main analysis contains different numbers of frames across speeds, the final 5-s window was aligned to the end of the selected walking segment rather than forced to a fixed 10-15 s interval for every speed.

C.2. Results

Across all speeds and all 5-s windows, the window-level SDR partitions remained highly similar to the full-segment $h = 0.80$ reference partition. The mean ARI was 0.807 ± 0.103, and the mean NMI was 0.881 ± 0.061. The mean Jaccard overlap of the five major SDRs was 0.811 ± 0.099, and the mean centroid displacement of matched major SDRs was 26.1 mm. These results indicate that the principal SDR organization was preserved across 5-s temporal windows.

Adjacent-window similarity was also high, with mean adjacent-window ARI = 0.839 and mean adjacent-window NMI = 0.903. The beginning-to-end comparison showed mean ARI = 0.803 ± 0.040 and mean NMI = 0.874 ± 0.043 between the first and final 5-s windows. Thus, the main bilateral and spinal/midline SDR organization did not undergo a large temporal reconfiguration between the beginning and end of the selected walking segment.

The spatial maps further support this conclusion. The dominant left and right SDRs and the spinal midline SDR remained visually identifiable in the early, middle, and late windows. Smaller peripheral SDRs, isolated markers, and singleton/tiny communities showed greater window-to-window variation.

Representative full-segment, early-window, middle-window, and late-window SDR maps are shown in Fig F in S1 Text. Window-level temporal-stability metrics across all 5-s windows are shown in Fig G in S1 Text.

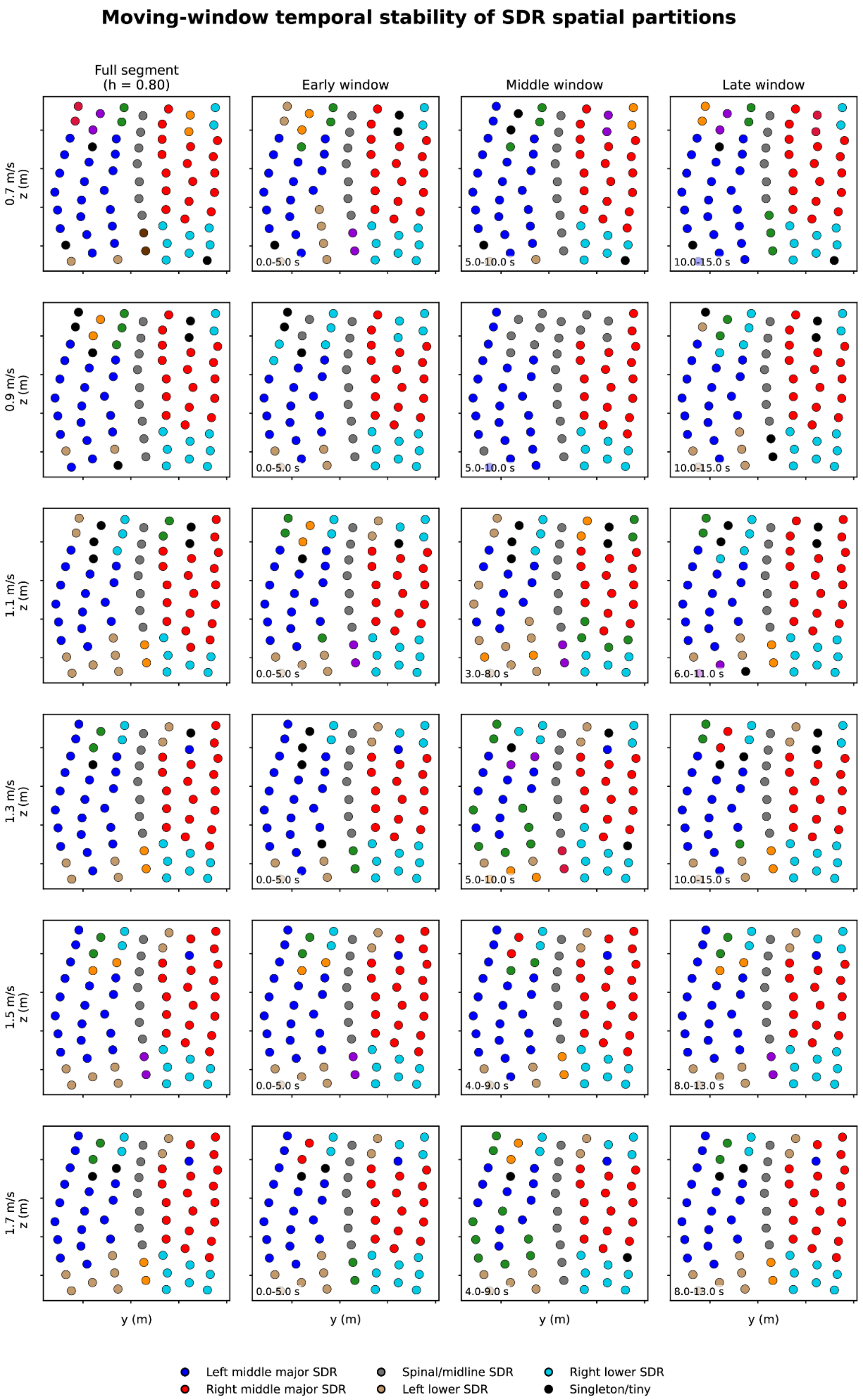

Fig F. Moving-window temporal stability of SDR spatial partitions. Full-segment $h = 0.80$ SDR partitions are compared with representative 5-s moving-window partitions for each walking speed. For each speed, the full-segment partition is shown together with an early, middle, and late 5-s window. The final 5-s window was aligned to the end of the same selected walking segment used in the main analysis. The principal bilateral and spinal/midline SDRs remained identifiable across temporal windows, whereas smaller peripheral and singleton or tiny communities showed greater window-to-window variability.

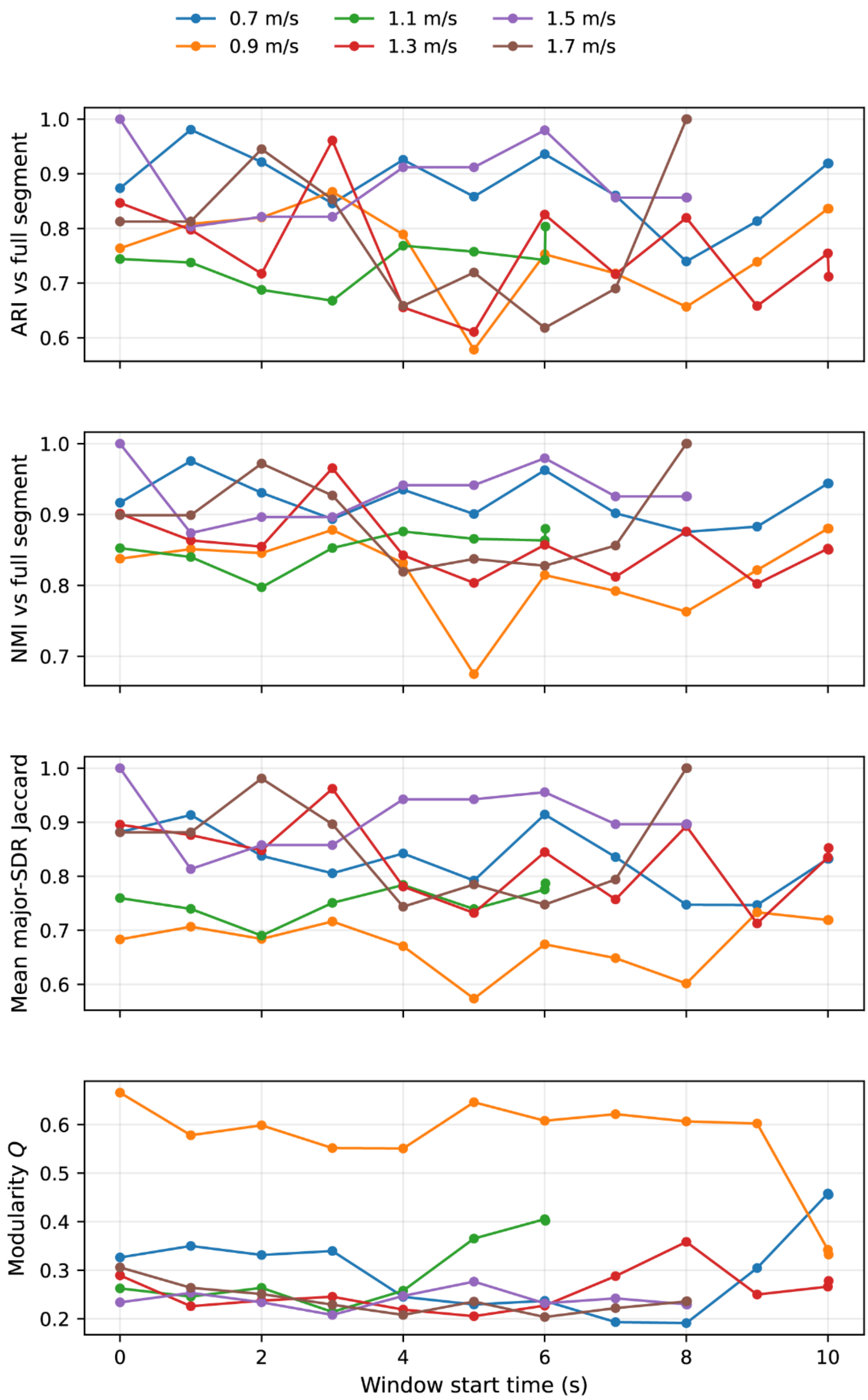

Fig G. Temporal stability metrics from 5-s moving windows. Window-level SDR partitions were compared with the corresponding full-segment $h = 0.80$ reference partition using ARI, NMI, mean major-SDR Jaccard overlap, and modularity Q. Across walking speeds, the window-level partitions remained highly similar to the full-segment reference, supporting the interpretation of the full-segment SDR partition as a stable principal or time-averaged deformation organization.
