# Supplementary material for: Network analysis of surface deformation reveals trunk modularity and synchronization during gait
Source: PLoS Comput Biol. 2026 Jul 24;22(7):e1014388. doi: 10.1371/journal.pcbi.1014388 (PMC13524338; doi:10.1371/journal.pcbi.1014388)
Supplement: Supplementary file 2 [file pcbi.1014388.s002.docx]

**Response to Reviewers**

**Additional Editor Comments:** The reviewers think the work of interest to the journal and recommend some revisions, particularly regarding the sample size of the experiment. Please address them constructively in your revised manuscript.

**Response:** We appreciate the Editor for providing an opportunity to revise our manuscript based on the reviewers’ constructive and positive comments. We have addressed each point raised by the reviewers in detail below, with particular attention to the sample-size limitation, the robustness of the network analysis, the marker-density sensitivity analysis, and the temporal stability of the SDR partitions. We have also revised the manuscript to clarify the proof-of-concept scope of the study and to avoid overgeneralized claims based on the single-participant dataset.

**Reviewers' comments**

Reviewer #1

**Comment 1.1**

This manuscript presents an innovative methodological framework integrating high-density surface topography (utilizing 95 reflective markers) with complex network analysis to decode the dynamic deformation of the human trunk during gait. By modeling the dorsal skin as a dynamic continuum and applying community detection algorithms, the authors identified Synchronous Deformation Regions (SDRs) which are robust under different walking speeds (0.7 m/s to 1.7 m/s). Furthermore, the study uncovered long-range synchronization between the scapular and contralateral pelvic regions at speeds higher than 1.1 m/s. Projecting multi-dimensional, non-linear musculoskeletal synergies onto a 2D topological network is a highly effective approach. This methodology has significant potential to inform the design and control strategies of spinal exoskeletons and enhance precision rehabilitation assessments. The mathematical modeling is sound. However, the small sample size of only one subject limit the generalizability of the conclusions making this work more appropriate as a feasibility or pilot study. To solidify the manuscript for publication, the robustness of certain network parameters and the limitation of experiments require further clarification. In addition, some specific comments are listed as follows.

**Response:** We appreciate the reviewer’s constructive and positive comments, as well as the recognition of the methodological contribution of integrating high-density surface topography with complex network analysis to characterize trunk deformation during gait. We agree that the single-participant design limits the generalizability of the conclusions and that the robustness of key network parameters should be clarified.

In the revised manuscript, we clarified the scope of the study as a proof-of-concept analysis, rewrote the “Limitations and future work” and “Conclusion” sections to avoid overgeneralized claims, and softened statements implying population-level validity or a clinically validated biomarker. We also added a Supporting Information text containing threshold-sensitivity analysis to evaluate the robustness of SDR identification. Our point-by-point responses are provided below. Line numbers refer to the revised manuscript with tracked changes.

**Comment 1.2**

The construction of the signed correlation network relies on a strict absolute threshold of to prune weak edges. Such hard thresholding can fundamentally alter the topology and the resulting modularity partition (reported as). How do the spatial boundaries of the SDRs shift if or is applied? Confirming the topological robustness against this hyperparameter will significantly strengthen the mathematical rigor of the study.

**Response:** We thank the reviewer for raising this important methodological point. We agree that hard thresholding can influence graph sparsity, connectedness, modularity, and the resulting SDR partition. To address this concern, we added a threshold-sensitivity analysis in the Supporting Information.

Specifically, we repeated the complete SDR-identification pipeline for five absolute correlation thresholds, $h=0.70, 0.75, 0.80, 0.85,$ and $0.90$, across all six walking speeds. The $h=0.80$ partition was used as the reference because this was the threshold used in the main analysis. For each threshold and walking speed, we quantified network-level metrics, including edge count, edge density, positive and negative edge counts, connected components, modularity $Q$, and the number of SDRs. We also compared each partition with the $h=0.80$ reference using adjusted Rand index (ARI), normalized mutual information (NMI), Jaccard overlap of matched major SDRs, centroid displacement, and relative area change.

The results showed that thresholding changed network sparsity as expected. Mean edge density decreased from 0.278 at $h=0.70$ to 0.188 at $h=0.80$ and 0.078 at $h=0.90$. Mean modularity $Q$ increased from 0.260 at $h=0.70$ to 0.338 at $h=0.80$ and 0.567 at $h=0.90$, consistent with progressive edge pruning and graph fragmentation. However, the principal SDR organization remained stable relative to the $h=0.80$ reference partition. Across all non-reference thresholds, ARI ranged from 0.564 to 0.968 and NMI ranged from 0.696 to 0.969. For moderate threshold perturbations, $h=0.75$ and $h=0.85$, mean ARI was 0.863, mean NMI was 0.910, and mean Jaccard overlap of the five major SDRs was 0.841.

The two dominant lateral SDRs were particularly stable. Across non-reference thresholds, the two largest SDRs showed mean Jaccard overlaps of 0.866, 0.886, 0.939, and 0.818 at $h=0.70, 0.75, 0.85$, and $0.90$, respectively. Their mean centroid displacements were 9.7 mm, 11.9 mm, 5.8 mm, and 18.3 mm at these thresholds, respectively. These results indicate that the principal bilateral thoracolumbar SDRs are not an artifact of the specific $h=0.80$ threshold.

At the most stringent threshold, $h=0.90$, weaker long-range and peripheral edges were pruned more aggressively, resulting in lower mean ARI (0.700), lower mean NMI (0.822), and lower mean major-SDR Jaccard overlap (0.649). We have therefore revised the manuscript to state that the major bilateral and spinal SDRs were robust to reasonable threshold perturbations, whereas smaller peripheral SDRs should be interpreted more cautiously.

**Changes made:** We added the complete threshold-sensitivity analysis to Section A of S1 Text, including Fig A, Fig B, Table A in S1 Text.

In the manuscript, we added concise cross-references in Section 2.3 (Line 142-143) and 5.3.4 (Line 396-400) to state that the robustness of SDR identification to threshold selection was evaluated in the Supporting Information.

**Comment 1.3**

When discussing the Posterior Oblique Subsystem and the myofascial 'Spiral Line', please explicitly connect how the discrete muscle forces mathematically translate into the positive correlation edges observed in the mid-thoracic topological network.

**Response:** We thank the reviewer for highlighting the need to bridge the gap between discrete muscle activation and topological network edges. Anatomically, muscles such as the latissimus dorsi and gluteus maximus do not act as isolated point masses; their contraction forces are transmitted through the continuous thoracolumbar fascia. This fascial tension creates a macroscopic, continuous strain field across the overlying dorsal skin. Consequently, markers situated within this specific region undergo synchronous, in-phase stretching and compression during the gait cycle. In our mathematical framework, this localized phase consistency translates directly into high positive rank correlation coefficients ($\rho_{ij}>0.8$), which the Louvain algorithm subsequently identifies as tightly connected positive edges, ultimately grouping them into a distinct mid-thoracic topological module.

**Changes made:** In the revised manuscript, we have clarified this mapping mechanism in Section 3.1 (Line 183-186).

**Comment 1.4**

The distinct grey cluster along the spinal midline is identified as a kinematic boundary. Please clarify if the stability of this topological region is primarily governed by the rigid-body dynamics of the underlying spinous processes rather than the viscoelastic deformation of the adjacent paraspinal skin.

**Response:** The reviewer raises an excellent point regarding the boundary conditions of this dynamic system. We concur that the topological stability of the midline cluster is predominantly governed by the rigid-body kinematics of the underlying spine. Anatomically, the skin and superficial fascia along the spinal midline is firmly anchored to the supraspinous ligaments and spinous processes. This mechanical constraint severely limits its ability to undergo the large viscoelastic lateral stretching observed in the paraspinal regions, which are actively driven by underlying muscle contractions. Because its temporal deformation profile is constrained by this rigid-body dynamic, it fails to form strong positive correlations with the lateral paraspinal markers, leading to its emergence as an isolated, stable kinematic boundary in our network model.

**Changes made:** In the revised manuscript, we have clarified this mapping mechanism in Section 3.1 (Line 162-165).

**Comment 1.5**

In Figure 4, the baseline value is set to 1.00. Please ensure it is explicitly stated in the figure legend that this represents the normalized average calculated across all tested walking speeds to prevent any misinterpretation of the geometric variance.

**Response:** Thank you for this suggestion. We agree that this definition should be maximally explicit. We have revised the Figure 4 caption to state more clearly that the dashed line at 1.00 denotes the within-subject mean across all six tested walking speeds for the corresponding geometric parameter.

**Changes made:** In the revised manuscript, we have clarified this in the caption of Figure 4.

**Comment 1.6**

Grammatical mistakes and typos still exist. In Abstract, the phrase "addressing prevalence disorders like low back pain" is not appropriate. Consider changing "prevalence disorders" to "prevalent disorders" for better clarity. In Section 3.1, line 191, there is a missing numerical value “the superior angle of the respective contralateral scapulae at speeds ≥ m/s”. In Section 3.3, the section title contains a spelling error “Comparision”.

**Response:** We thank the reviewer for identifying these wording issues. We have corrected “prevalence disorders” to “prevalent disorders” in the Abstract, corrected the missing speed value $1.1$m/s in Section 3.1, changed “Comparision” to “Comparison” in the Section 3.3 title, and performed an additional language-polishing pass throughout the manuscript.

Reviewer #2

**Comment 2.1**

This paper proposes the use of a large number of force markers to measure surface tension while a human subject walks on a treadmill at different speeds. Through correlation and network analyses, the authors identify local clusters across the sampling points under different gait conditions. The study provides a potentially effective approach for capturing detailed surface-tension patterns and estimating trunk deformation during walking. The manuscript is generally well written, and the figures are of high quality. I have the following comments before recommending publication:

**Response:** We appreciate the reviewer’s constructive and positive comments and the recognition that the proposed approach may help capture detailed trunk surface-deformation patterns during walking. We also appreciate the opportunity to clarify the terminology and methodological scope of the study.

In response to the reviewer’s comments, we added a Supporting Information text containing marker-density sensitivity analysis and a 5-s moving-window temporal stability analysis. In the revised manuscript, we clarified the scope of the study as a proof-of-concept analysis, rewrote the “Limitations and future work” and “Conclusion” sections to avoid overgeneralized claims, and softened statements implying population-level validity or a clinically validated biomarker. We also clarified the roles of motion-capture and GRF data in the analysis. Our point-by-point responses are provided below. Line numbers refer to the revised manuscript with tracked changes.

**Comment 2.2**

Is there a specific reason for choosing such a large number of sensors (95) for this task? Would it be possible to reach similar conclusions with, for example, half that number? In addition, are all sampling channels synchronized? If not, could any delay between channels affect the analysis?

**Response:** We thank the reviewer for raising this important methodological point. The 95-marker configuration was selected because the local deformation variable is a neighborhood area observable. Each analyzed marker requires neighboring markers to define a local surface patch. Reducing marker density therefore changes not only the number of network nodes but also the spatial scale at which surface deformation is estimated. A sparse marker set may still capture coarse left-right trunk deformation, but it may fail to preserve local SDR boundaries, spinal midline organization, and smaller boundary-sensitive SDRs.

To address the reviewer’s question directly, we added a marker-density sensitivity analysis using two regular column-reduced layouts in the revised manuscript and Supporting Information.

Since the original marker field forms a staggered lattice, we tested two complementary reduced configurations based on odd/even column retention. The odd-column layout retained 55 markers, including 27 analyzed markers and 28 boundary markers. The even-column layout retained 40 analyzed markers and additionally retained the original 28 boundary markers, yielding 68 retained markers in total. These boundary markers were not included as network nodes; they were used only to define local neighborhoods.

We also revised the reduced-density neighborhood area calculation, since removing markers breaks the original full-density neighborhood structure. We defined fixed neighborhoods based on the original marker lattice. For the odd-column layout, each analyzed marker used the four surrounding retained markers to form four local triangles. For the even-column layout, each analyzed marker used a fixed ring of surrounding retained markers, including original boundary markers where necessary. (See Figure C in S1 Text for details.) The local neighborhood area was computed as the sum of the surrounding triangle areas, consistent with the full-density local-area definition. The same fixed neighborhood topology was reused for all walking speeds.

The reduced-density results showed moderate but incomplete agreement with the full-density $h=0.80$ reference partition. Across walking speeds, the odd-column layout yielded mean ARI = 0.550, mean NMI = 0.761, mean major-SDR Jaccard overlap = 0.654, and mean centroid displacement = 40.3 mm. The even-column layout with boundary/support markers yielded mean ARI = 0.594, mean NMI = 0.675, mean major-SDR Jaccard overlap = 0.628, and mean centroid displacement = 57.2 mm. Thus, both reduced layouts preserved part of the coarse bilateral deformation organization, but neither reliably reproduced the detailed full-density SDR partition. These results support the use of the 95-marker configuration for the present high-resolution proof-of-concept analysis. The dense marker layout was not simply redundant; it provided the spatial support required to define local surface neighborhoods and to resolve smaller or boundary-sensitive SDRs. Future simplified clinical marker sets may be feasible, but they should be optimized and validated separately rather than assumed from simple removal.

Regarding synchronization, all marker trajectories were reconstructed within the same Vicon optical motion-capture frame sequence and therefore shared a common sampling clock and timestamp. There was no marker-specific channel delay among the reflective markers. The TRC marker trajectories were sampled at 100 Hz. The GRF file was sampled at 1000 Hz, with ten force subframes for each marker frame, consistent with synchronized force-plate acquisition relative to the motion-capture frame index. Importantly, the SDR network was constructed exclusively from marker-derived local surface-area time series; GRF data were not used to compute the Spearman correlation matrix, construct the network, perform community detection, or assign SDR labels. Therefore, any marker-to-GRF timing offset would not affect the SDR network analysis.

**Changes made:** We added a marker-density sensitivity analysis to Section B of S1 Text. The spatial comparison is shown in Figure D in S1 Text. The corresponding quantitative similarity metrics, including ARI, NMI, major-SDR Jaccard overlap, and centroid displacement, are shown in Figure E in S1 Text.

In the manuscript, we added concise cross-references in Section 2.3 (Line 142-143) and 5.3.4 (Line 396-400) to state that the robustness of SDR identification to marker density was evaluated in the Supporting Information. We also revised Section 5.2 (Line 332-336) to clarify that the sampling points were passive reflective markers. All marker trajectories shared the same motion-capture frame sequence, so there was no marker-specific delay among the marker trajectories.

**Comment 2.3**

From my reading, it appears that there is only one participant and one trial for each walking speed. I understand that this is not an easy experiment to conduct. However, a single example may not be sufficient to justify the generality of either the method or the conclusions.

**Response:** We fully agree with the reviewer. The present dataset consists of one healthy participant and one continuous trial for each walking speed. Therefore, it cannot support population-level generalization, inter-subject variability estimation, test-retest reliability assessment, or clinical diagnostic validation.

The primary aim of this study is not to establish population-level biological generality or clinical validity, but to introduce and technically demonstrate a framework integrating body surface topography with network analysis for characterizing dorsal surface deformation during gait. Within this limited scope, each selected walking segment contained multiple gait cycles, which allowed us to estimate within-trial marker-correlation structure and examine whether the proposed SDR identification pipeline behaved consistently across walking speeds.

We have revised the manuscript to make this scope explicit. In particular, we rewrote Section 3.5 “Limitations and future work” and Section 4 “Conclusion” to state that the identified SDRs should be interpreted as within-participant proof-of-concept patterns rather than population-level anatomical modules or validated clinical biomarkers. We also made targeted wording changes in the Abstract, Author Summary, Introduction, and Discussion to avoid implying that the current single-participant dataset establishes general biological validity, diagnostic utility, or a validated clinical biomarker. We now state that larger cohorts, repeated trials, and clinical populations are required to evaluate inter-subject variability, test-retest reliability, and diagnostic relevance.

**Changes made:** We rewrote Section 3.5 “Limitations and future work” (Line 259-280) and Section 4 “Conclusion” (Line 282-295) to explicitly acknowledge the single-participant, single-trial design and to clarify the scope of inference. We also made targeted wording changes in the Abstract, Author Summary, Introduction (Line 40-42) and Discussion (Line 147, 152-154, 237-240, 242-245, 254-257) to avoid implying that the current single-participant dataset establishes population-level generality or a validated clinical biomarker.

**Comment 2.4**

It seems that the full 15-second dataset was pooled for the time-series analysis. Have the authors considered using a moving window, for example 5 seconds, to examine the temporal dynamics of clustering? This could reveal whether the cluster structure changes between the beginning and the end of walking.

**Response:** We thank the reviewer for this important suggestion. We agree that using the full walking segment produces a time-averaged correlation network and may obscure temporal changes in SDR organization. To address this concern, we added a 5-s moving-window temporal-stability analysis in the revised manuscript and Supporting Information.

For each walking speed, we used the same selected walking segment as in the main analysis. The marker-derived normalized neighborhood area time series were divided into 5-s windows with a 1-s step. At the 100-Hz marker sampling rate, each window contained 500 frames. For each window, we repeated the complete SDR-identification pipeline, including Spearman correlation calculation, FDR filtering, construction of the signed network at $h=0.80$, modularity-based community detection, and sign-based subdivision into positively connected SDRs. Each window-level partition was compared with the corresponding full-segment $h=0.80$ reference partition using ARI, NMI, Jaccard overlap of the five major SDRs, and centroid displacement. We also computed adjacent-window similarity and directly compared the first and final 5-s windows.

The moving-window analysis showed that the principal SDR organization was temporally stable. Across all 5-s windows and walking speeds, mean ARI relative to the full-segment reference was 0.807 ± 0.103, mean NMI was 0.881 ± 0.061, mean major-SDR Jaccard overlap was 0.811 ± 0.099, and mean centroid displacement of matched major SDRs was 26.1 mm. Adjacent-window similarity was also high, with mean adjacent-window ARI = 0.839 and mean adjacent-window NMI = 0.903. The beginning-to-end comparison showed mean ARI = 0.803 ± 0.040 and mean NMI = 0.874 ± 0.043 between the first and final 5-s windows. These results indicate that the dominant bilateral and spinal midline SDR organization was not an artifact of pooling the full segment.

We also observed that smaller peripheral SDRs and singleton or tiny communities showed greater window-to-window variability. We therefore revised the manuscript to clarify that the full-segment SDR partition should be interpreted as a stable principal or time-averaged representation of dorsal-surface deformation organization, while smaller peripheral clusters should be interpreted with greater caution.

**Changes made:** We added a moving-window temporal-stability analysis to Section C of S1 Text. Representative full-segment, early-window, middle-window, and late-window SDR maps are shown in Fig F in S1 Text. Window-level temporal-stability metrics across all 5-s windows are shown in Fig G in S1 Text.

In the manuscript, we added concise cross-references in Section 2.3 (Line 142-143) and 5.3.4 (Line 396-400) to state that the robustness of SDR identification to 5-s moving windows was evaluated in the Supporting Information.

**Comment 2.5**

The authors also collected motion-capture and force data during walking, but I do not see much analysis of these measurements. It would be helpful to clarify how these data were used, or to include additional analysis if relevant.

**Response:** Thank you for pointing out this ambiguity. The motion-capture data were not auxiliary measurements; they were the primary data source for the SDR analysis. The normalized neighborhood-area time series were computed from the three-dimensional trajectories of the dorsal reflective markers. In contrast, the GRF data recorded by the force-sensing treadmill were used only for gait-event annotation, including heel strike and toe-off, and for aligning deformation time series with gait phases, as visualized in the vertical indicator lines in Figure 1(a). GRF data were not used to compute the Spearman correlation matrix, construct the marker network, perform community detection, or assign SDR labels. We did not perform inverse dynamics or joint-torque analysis because the focus of this study was marker-derived dorsal surface deformation.

**Changes made:** We revised Section 5.2 (Line 337-339) to clarify the usage of our data.

**Minor comments:**
Line 316: Please add a space between “treadmill.” and “The”.
Line 319: “irrelevant noise5.” should be corrected to “irrelevant noise.”

**Response:** We thank the reviewer for identifying these wording issues. We have added a space between “treadmill.” and “The” and deleted “5”.
