# Supplementary material for: Network analysis of surface deformation reveals trunk modularity and synchronization during gait
Source: PLoS Comput Biol. 2026 Jul 24;22(7):e1014388. doi: 10.1371/journal.pcbi.1014388 (PMC13524338; doi:10.1371/journal.pcbi.1014388)
Supplement: Peer review history

# Peer review: Decision Letter 0

**DOI:** 10.1371/journal.pcbi.1014388.r001

**Authors:** Feng Fu

8 Apr 2026

PCOMPBIOL-D-26-00334

Network analysis of surface deformation reveals trunk modularity and synchronization during gait

PLOS Computational Biology

Dear Dr. Wang,

Thank you for submitting your manuscript to PLOS Computational Biology. After careful consideration, we feel that it has merit but does not fully meet PLOS Computational Biology's publication criteria as it currently stands. Therefore, we invite you to submit a revised version of the manuscript that addresses the points raised during the review process.

Please submit your revised manuscript by Jun 08 2026 11:59PM. If you will need more time than this to complete your revisions, please reply to this message or contact the journal office at ploscompbiol@plos.org. When you're ready to submit your revision, log on to https://www.editorialmanager.com/pcompbiol/ and select the 'Submissions Needing Revision' folder to locate your manuscript file.

Please include the following items when submitting your revised manuscript:

* A letter that responds to each point raised by the editor and reviewer(s). You should upload this letter as a separate file labeled 'Response to Reviewers'. This file does not need to include responses to formatting updates and technical items listed in the 'Journal Requirements' section below.

* A marked-up copy of your manuscript that highlights changes made to the original version. You should upload this as a separate file labeled 'Revised Manuscript with Track Changes'.

* An unmarked version of your revised paper without tracked changes. You should upload this as a separate file labeled 'Manuscript'.

If you would like to make changes to your financial disclosure, competing interests statement, or data availability statement, please make these updates within the submission form at the time of resubmission. Guidelines for resubmitting your figure files are available below the reviewer comments at the end of this letter.

We look forward to receiving your revised manuscript.

Kind regards,

Feng Fu

Section Editor

PLOS Computational Biology

Feng Fu

Section Editor

PLOS Computational Biology

**Additional Editor Comments:**

The reviewers think the work of interest to the journal and recommend some revisions, particularly regarding the sample size of the experiment. Please address them constructively in your revised manuscript.

**Journal Requirements:**

If the reviewer comments include a recommendation to cite specific previously published works, please review and evaluate these publications to determine whether they are relevant and should be cited. There is no requirement to cite these works unless the editor has indicated otherwise.

1) We ask that a manuscript source file is provided at Revision. Please upload your manuscript file as a .doc, .docx, .rtf or .tex. If you are providing a .tex file, please upload it under the item type u2018LaTeX Source Fileu2019 and leave your .pdf version as the item type u2018Manuscriptu2019.

2) Please upload all main figures as separate Figure files in .tif or .eps format. For more information about how to convert and format your figure files please see our guidelines:

https://journals.plos.org/ploscompbiol/s/figures

3) Please amend your detailed Financial Disclosure statement. This is published with the article. It must therefore be completed in full sentences and contain the exact wording you wish to be published.

1) State the initials, alongside each funding source, of each author to receive each grant. For example: "This work was supported by the National Institutes of Health (####### to AM; ###### to CJ) and the National Science Foundation (###### to AM)."

2) State what role the funders took in the study. If the funders had no role in your study, please state: "The funders had no role in study design, data collection and analysis, decision to publish, or preparation of the manuscript."

3) If any authors received a salary from any of your funders, please state which authors and which funders..

If you did not receive any funding for this study, please simply state: u201cThe authors received no specific funding for this work.u201d

4) Some material included in your submission may be copyrighted. According to PLOS's copyright policy, authors who use figures or other material (e.g., graphics, clipart, maps) from another author or copyright holder must demonstrate or obtain permission to publish this material under the Creative Commons Attribution 4.0 International (CC BY 4.0) License used by PLOS journals. Please closely review the details of PLOS's copyright requirements here: PLOS Licenses and Copyright. If you need to request permissions from a copyright holder, you may use the PLOS Content Copyright Permission Form Please respond directly to this email and provide any known details concerning your material's license terms and permissions required for reuse, even if you have not yet obtained copyright permissions or are unsure of your material's copyright compatibility. Once you have responded and addressed all other outstanding technical requirements, you may resubmit your manuscript within Editorial Manager. Potential Copyright Issues:

i) Please confirm (a) that you are the photographer of 5i, or (b) provide written permission from the photographer to publish the photo(s) under our CC BY 4.0 license.

ii) Figures 3, 5a, 5ii, 6a, and 6e. Please confirm whether you drew the images / clip-art within the figure panels by hand. If you did not draw the images, please provide (a) a link to the source of the images or icons and their license / terms of use; or (b) written permission from the copyright holder to publish the images or icons under our CC BY 4.0 license. Alternatively, you may replace the images with open source alternatives. See these open source resources you may use to replace images / clip-art: - https://commons.wikimedia.org - https://openclipart.org/.

**Reviewers' comments:**

Reviewer's Responses to Questions

**Comments to the Authors:**

**Please note here if the review is uploaded as an attachment.**

Reviewer #1: This manuscript presents an innovative methodological framework integrating high-density surface topography (utilizing 95 reflective markers) with complex network analysis to decode the dynamic deformation of the human trunk during gait. By modeling the dorsal skin as a dynamic continuum and applying community detection algorithms, the authors identified Synchronous Deformation Regions (SDRs) which are robust under different walking speeds (0.7 m/s to 1.7 m/s). Furthermore, the study uncovered long-range synchronization between the scapular and contralateral pelvic regions at speeds higher than 1.1 m/s. Projecting multi-dimensional, non-linear musculoskeletal synergies onto a 2D topological network is a highly effective approach. This methodology has significant potential to inform the design and control strategies of spinal exoskeletons and enhance precision rehabilitation assessments. The mathematical modeling is sound. However, the small sample size of only one subject limit the generalizability of the conclusions making this work more appropriate as a feasibility or pilot study. To solidify the manuscript for publication, the robustness of certain network parameters and the limitation of experiments require further clarification. In addition, some specific comments are listed as follows.

1. The construction of the signed correlation network relies on a strict absolute threshold of to prune weak edges. Such hard thresholding can fundamentally alter the topology and the resulting modularity partition (reported as). How do the spatial boundaries of the SDRs shift if or is applied? Confirming the topological robustness against this hyperparameter will significantly strengthen the mathematical rigor of the study.

2. When discussing the Posterior Oblique Subsystem and the myofascial 'Spiral Line', please explicitly connect how the discrete muscle forces mathematically translate into the positive correlation edges observed in the mid-thoracic topological network.

3. The distinct grey cluster along the spinal midline is identified as a kinematic boundary. Please clarify if the stability of this topological region is primarily governed by the rigid-body dynamics of the underlying spinous processes rather than the viscoelastic deformation of the adjacent paraspinal skin.

4. In Figure 4, the baseline value is set to 1.00. Please ensure it is explicitly stated in the figure legend that this represents the normalized average calculated across all tested walking speeds to prevent any misinterpretation of the geometric variance.

5. Grammatical mistakes and typos still exist. In Abstract, the phrase "addressing prevalence disorders like low back pain" is not appropriate. Consider changing "prevalence disorders" to "prevalent disorders" for better clarity. In Section 3.1, line 191, there is a missing numerical value “the superior angle of the respective contralateral scapulae at speeds ≥ m/s”. In Section 3.3, the section title contains a spelling error “Comparision”.

Reviewer #2: This paper proposes the use of a large number of force markers to measure surface tension while a human subject walks on a treadmill at different speeds. Through correlation and network analyses, the authors identify local clusters across the sampling points under different gait conditions. The study provides a potentially effective approach for capturing detailed surface-tension patterns and estimating trunk deformation during walking. The manuscript is generally well written, and the figures are of high quality. I have the following comments before recommending publication:

1. Is there a specific reason for choosing such a large number of sensors (95) for this task? Would it be possible to reach similar conclusions with, for example, half that number? In addition, are all sampling channels synchronized? If not, could any delay between channels affect the analysis?

2. From my reading, it appears that there is only one participant and one trial for each walking speed. I understand that this is not an easy experiment to conduct. However, a single example may not be sufficient to justify the generality of either the method or the conclusions.

3. It seems that the full 15-second dataset was pooled for the time-series analysis. Have the authors considered using a moving window, for example 5 seconds, to examine the temporal dynamics of clustering? This could reveal whether the cluster structure changes between the beginning and the end of walking.

4. The authors also collected motion-capture and force data during walking, but I do not see much analysis of these measurements. It would be helpful to clarify how these data were used, or to include additional analysis if relevant.

Minor comments:

Line 316: Please add a space between “treadmill.” and “The”.

Line 319: “irrelevant noise5.” should be corrected to “irrelevant noise.”

**********

**Have the authors made all data and (if applicable) computational code underlying the findings in their manuscript fully available?**

The PLOS Data policy requires authors to make all data and code underlying the findings described in their manuscript fully available without restriction, with rare exception (please refer to the Data Availability Statement in the manuscript PDF file). The data and code should be provided as part of the manuscript or its supporting information, or deposited to a public repository. For example, in addition to summary statistics, the data points behind means, medians and variance measures should be available. If there are restrictions on publicly sharing data or code —e.g. participant privacy or use of data from a third party—those must be specified.

Reviewer #1: Yes

Reviewer #2: None

**********

PLOS authors have the option to publish the peer review history of their article (what does this mean?). If published, this will include your full peer review and any attached files.

If you choose “no”, your identity will remain anonymous but your review may still be made public.

**Do you want your identity to be public for this peer review?**  For information about this choice, including consent withdrawal, please see our Privacy Policy.

Reviewer #1: No

Reviewer #2: No

**Figure resubmission:**

**Reproducibility:**

To enhance the reproducibility of your results, we recommend that authors of applicable studies deposit laboratory protocols in protocols.io, where a protocol can be assigned its own identifier (DOI) such that it can be cited independently in the future. Additionally, PLOS ONE offers an option to publish peer-reviewed clinical study protocols. Read more information on sharing protocols at https://plos.org/protocols?utm_medium=editorial-email&utm_source=authorletters&utm_campaign=protocols

# Author comment: Author response to Decision Letter 1

**DOI:** 10.1371/journal.pcbi.1014388.r002

15 May 2026

Attachment Submitted filename: reply_to_comments.docx

# Peer review: Decision Letter 1

**DOI:** 10.1371/journal.pcbi.1014388.r003

**Authors:** Feng Fu

1 Jun 2026

Dear Professor Wang,

We are pleased to inform you that your manuscript 'Network analysis of surface deformation reveals trunk modularity and synchronization during gait' has been provisionally accepted for publication in PLOS Computational Biology.

Before your manuscript can be formally accepted you will need to complete some formatting changes, which you will receive in a follow up email. A member of our team will be in touch with a set of requests.

Please note that your manuscript will not be scheduled for publication until you have made the required changes, so a swift response is appreciated.

IMPORTANT: The editorial review process is now complete. PLOS will only permit corrections to spelling, formatting or significant scientific errors from this point onwards. Requests for major changes, or any which affect the scientific understanding of your work, will cause delays to the publication date of your manuscript.

Should you, your institution's press office or the journal office choose to press release your paper, you will automatically be opted out of early publication. We ask that you notify us now if you or your institution is planning to press release the article. All press must be co-ordinated with PLOS.

Thank you again for supporting Open Access publishing; we are looking forward to publishing your work in PLOS Computational Biology.

Best regards,

Feng Fu

Section Editor

PLOS Computational Biology

***********************************************************

Reviewer's Responses to Questions

**Comments to the Authors:**

**Please note here if the review is uploaded as an attachment.**

Reviewer #1: The authors have addressed my comments accordingly and I support the publication of the paper in the journal.

Reviewer #2: All of my questions have now been addressed, Thanks.

**********

**Have the authors made all data and (if applicable) computational code underlying the findings in their manuscript fully available?**

The PLOS Data policy requires authors to make all data and code underlying the findings described in their manuscript fully available without restriction, with rare exception (please refer to the Data Availability Statement in the manuscript PDF file). The data and code should be provided as part of the manuscript or its supporting information, or deposited to a public repository. For example, in addition to summary statistics, the data points behind means, medians and variance measures should be available. If there are restrictions on publicly sharing data or code —e.g. participant privacy or use of data from a third party—those must be specified.

Reviewer #1: None

Reviewer #2: None

**********

PLOS authors have the option to publish the peer review history of their article (what does this mean?). If published, this will include your full peer review and any attached files.

If you choose “no”, your identity will remain anonymous but your review may still be made public.

**Do you want your identity to be public for this peer review?**  For information about this choice, including consent withdrawal, please see our Privacy Policy.

Reviewer #1: No

Reviewer #2: No

# Editor report: Acceptance letter

**DOI:** 10.1371/journal.pcbi.1014388.r004

**Authors:** Feng Fu

PCOMPBIOL-D-26-00334R1

Network analysis of surface deformation reveals trunk modularity and synchronization during gait

Dear Dr Wang,

I am pleased to inform you that your manuscript has been formally accepted for publication in PLOS Computational Biology. Your manuscript is now with our production department and you will be notified of the publication date in due course.

The corresponding author will soon be receiving a typeset proof for review, to ensure errors have not been introduced during production. Please review the PDF proof of your manuscript carefully, as this is the last chance to correct any errors. Please note that major changes, or those which affect the scientific understanding of the work, will likely cause delays to the publication date of your manuscript.

Soon after your final files are uploaded, unless you have opted out, the early version of your manuscript will be published online. The date of the early version will be your article's publication date. The final article will be published to the same URL, and all versions of the paper will be accessible to readers.

For Research, Software, and Methods articles, you will receive an invoice from PLOS for your publication fee after your manuscript has reached the completed accept phase. If you receive an email requesting payment before acceptance or for any other service, this may be a phishing scheme. Learn how to identify phishing emails and protect your accounts at https://explore.plos.org/phishing.

Thank you again for supporting PLOS Computational Biology and open-access publishing. We are looking forward to publishing your work!

With kind regards,

Janani Seenivasan

PLOS Computational Biology | Carlyle House, Carlyle Road, Cambridge CB4 3DN | United Kingdom ploscompbiol@plos.org | Phone +44 (0) 1223-442824 | ploscompbiol.org | @PLOSCompBiol
